# Pathogenesis and molecular mechanisms of mutant TIE2-driven venous malformation

**DOI:** 10.1007/s10456-026-10088-y

**Published:** 2026-08-20

**Authors:** Lindsay J. Bischoff, Elisa Boscolo

**Affiliations:** 1 Division of Experimental Hematology and Cancer Biology, Cincinnati Children’s Hospital Medical Center, 3333 Burnet Avenue, Cincinnati, OH 45229 USA; 2 Department of Cancer Biology, University of Cincinnati College of Medicine, Cincinnati, OH USA; 3 Department of Pediatrics, University of Cincinnati College of Medicine, Cincinnati, OH USA

**Keywords:** Venous malformation, TIE2, PIK3CA, Vascular anomaly

## Abstract

Venous malformation (VM) is the most common subtype of vascular malformation. Due to the chronic nature of this disorder, VM patients face significant morbidity and complications throughout their lives. Current therapeutic options can be limited, but recent advances in understanding the cellular and molecular mechanisms that underly VM pathogenesis provide hope for the discovery of more effective targeted therapies. These advances arise from a greater understanding of the cellular effects of VM-causative mutations, which has been aided by the development of more advanced and physiologically relevant model systems. In this review, we begin by providing a brief overview of the clinical characteristics and genetic driver mutations of the most common subtypes of all vascular anomalies (including vascular tumors and vascular malformations), providing context for our more detailed discussion of these aspects of VM. We further summarize the current treatment options available for VM patients and the advancements that have been made in the use of targeted therapies for these patients. We further discuss recent advances in the development of model systems that can be used to study VM pathogenesis. Finally, we focus this review on the mechanisms that are downstream of mutant TIE2, discussing structural features of the receptor, TIE2 signaling pathways, and its roles in vascular physiology and VM pathology.

## Overview of vascular anomalies and venous malformation

Vascular anomalies encompass a group of disorders that are characterized by the abnormal development or growth of blood or lymphatic vessels. These span a range of presentation, from localized and quiescent lesions that appear as birthmarks to tumors that can grow and infiltrate surrounding tissue [1]. Standardized classification of vascular anomalies has emerged as an important requisite for correct differential diagnosis, treatment, and investigation of these disorders. This has been achieved by the International Society for the Study of Vascular Anomalies (ISSVA), which has published guidelines on the classification of vascular anomalies [2]. In this classification, vascular anomalies have been broadly defined as belonging to either the subgroup of vascular tumors or vascular malformations [2]. These major classifications, which are based on clinical and histological presentation, have implications for the pathobiology and cellular mechanisms that are thought to contribute to both. A summary of the subtypes of vascular anomalies discussed in this chapter is provided in Table 1. For representative clinical images of different subtypes of vascular anomalies, as compiled by ISSVA, the reader can refer to published comprehensive clinical [1] and radiological studies [3].

**Table 1:** Overview of vascular anomalies and genetics

Subcategory	Disorder	Clinical characteristics	Genes involved
*Vascular tumors*			
Benign	Infantile hemangioma (IH)	Cutaneous/subcutaneous and well-defined vascular masses. Distinct phases of proliferation (within weeks of birth to one year of age) and regression [6]	Non-inherited. Some risk factor variants/mutations in VEGF signaling components [44], but no definitive or widespread causative mutations
Locally aggressive	Tufted angioma (TA) /kaposiform hemangioendothelioma (KHE)	Locally aggressive vascular masses appearing in skin or soft tissues. Appear as firm plaques with ill-defined borders with red–purple coloration. Can lead to severe life-threatening consumptive coagulopathy (Kasabach-Merritt phenomenon) [7]	Non-inherited. Not fully elucidated, some activating mutations in *GNA14* [9]
Malignant	Epithelioid hemangioendothelioma (EHE)/angiosarcoma	Solid tumors consisting of abnormal endothelial cells that may or may not form vascular lumens. Highly proliferative and aggressive in nature and are capable of metastasis [11]	Non-inherited EHE: Gene fusions—*WWTR1-CAMTA1* and *YAP1-TFE3* Angiosarcoma: Hyperactivating mutations in oncogenes (*FLT4, KDR, MYC, KRAS*) or loss-of-function of tumor suppressors (*TP53*) [12]
*Vascular malformations*			
Fast flow	Sporadic arteriovenous malformation (AVM)	Diverse presentation, dependent on location. Consists of abnormal connections between artery and veins without an intervening capillary bed [13]	Somatic activating mutations in the RAS/MAPK/ERK pathway [14], including *KRAS*, *MAP2K1*, and *BRAF*
	Capillary-malformation arteriovenous malformation (CM-AVM)	Many small and/or single large superficial red patches (capillary malformations) that are typically present at birth. A subset of these patients also have AVMs [17]	Inherited loss-of-function mutations in *RASA1* [15] and *EPHB4* [16], affecting RAS/MAPK/ERK signaling
	Hereditary hemorrhagic telangiectasia (HHT)	Widespread appearance of superficial telangiectasias and lung, liver, and brain AVM [19]	Inherited loss-of-function mutations in the TGF-β signaling pathway, including *ENG*, *ACVRL*, and *SMAD4* [18]
Slow flow	Capillary malformation (CM)/Sturge Weber syndrome	Often superficial, red or pink flat birthmarks. Formed of dilated capillary vessels [20]. Neurological defects are also seen with Sturge Weber	Somatic activating mutations in *GNAQ* [21]
	Lymphatic malformation (LM) including: macrocystic and microcystic, general lymphatic anomaly (GLA), Kaposiform lymphangiomatosis (KLA), and Gorham-Stout disease (GSD)	Formation of abnormal lymphatic cysts (macrocystic or microcystic), diffuse proliferative lymphatic lesions (GLA/KLA), or lymphatic vessel overgrowth (GSD) [22]	Somatic activating mutations in *PIK3CA* [23] (macrocystic and microcystic and GLA), in *NRAS* (KLA) [24], and in *KRAS* (GSD) [25]
	Venous malformation (VM)	Compressible blue-purple masses in the skin or subcutaneous tissues formed of enlarged and structurally abnormal veins [36] (sporadic and inherited forms). Also includes disorders with clinically distinct venous anomalies including CCM [45] and GVM [46]	Hereditary or somatic activating mutations in *TIE2* [42, 47–50] (sporadic and inherited VM). Somatic activating mutations in *PIK3CA* [51–53] (sporadic VM). Loss-of-function mutations in *GMLN* (GVM) [46] and *KRIT1*, *CCM2*, *PDCD10* (CCM) [54, 55]

### Vascular tumors

Early classification attempts focused on understanding the underlying cellular pathology of the lesion. Initial observations were made that some lesions were more likely driven by high levels of endothelial cell (EC) proliferation [4]. These demonstrated signs of rapid postnatal growth and cellular proliferation, both via histology and clinical presentation [5], and were therefore termed as “vascular tumors”. Updates to the classification further divided the vascular tumors into benign, locally aggressive, and malignant subtypes, based on typical tumor growth patterns and ability to invade into surrounding tissues [1]. The most common type of vascular tumor is infantile hemangioma (IH), which is non-inherited, has an incidence of 4–5% of newborns and exemplifies the characteristics of a benign vascular tumor. They appear as well-defined red vascular masses that rapidly expand within weeks after birth and then spontaneously involute within the first years of life [6].

Locally aggressive vascular tumors are also non-inherited and include tufted angioma (TA) and kaposiform hemangioendothelioma (KHE), which are considered to be different clinical presentations along a spectrum of a single disease entity. They appear as firm red–purple plaque-like tumors in the skin or soft tissue that have ill-defined margins and can enlarge and invade local tissues, although metastasis does not occur [7]. The most severe complication of KHE and TA is a severe and life-threatening coagulopathy known as Kasabach–Merritt phenomenon (KMP). Although the cellular mechanisms that lead to KMP are unknown, it presents as thrombocytopenia and consumptive coagulopathy [7]. Recent experimental evidence suggests that the abnormal EC that line the diseased vessels may experience transcriptomic changes in coagulation pathways that could contribute to hemostatic changes, perhaps implicating an EC-centered mechanism by which KMP develops [8]. The molecular and genetic causes of TA/KHE have not been fully elucidated. However, some evidence suggests that activating genetic mutations play a role as hyperactivating mutations in *GNA14* (encoding the guanine nucleotide-binding protein subunit alpha-14) have been found in some patients with TA and KHE [9] and mutations in the related genes *GNAQ* and *GNA11* have been found in other types of vascular tumors [10].

Malignant vascular tumors are exceedingly rare and have oncogenic features, similar to other cancer types [11]. The two main types are epithelioid hemangioendothelioma (EHE) and angiosarcoma and these consist of tumors formed from vascular EC that have undergone somatic (non-inherited) oncogenic transformation due to genetic mutation and/or exposure to cancer-inducing agents, such as chemicals or radiation exposure (comprehensively reviewed in [12]).

### Vascular malformations

Whereas vascular tumors are thought to be the result of vascular overgrowth and EC proliferation, vascular malformations have historically been considered the result of errors in vascular morphogenesis [4]. Vascular malformations do not typically experience rapid growth phases (in contrast to vascular tumors), which was the first characteristic used to separate them from vascular tumors. They are further subdivided into fast-flow and slow-flow categories (based on the rate of blood flow through the diseased vessels), which are then specifically named by the type(s) of vessel that they are made of [2].

Fast-flow lesions typically contain an arterial component, such as arteriovenous malformations (AVM) or arteriovenous fistulas [2]. They also encompass hereditary AVM disorders, such as hereditary hemorrhagic telangiectasia (HHT) and capillary malformation-arteriovenous malformation syndrome (CM-AVM) [2]. AVMs consist of abnormal connections between arteries and veins, without an intervening capillary bed, resulting in the fast-flow shunting of blood between the artery and vein, which may cause hemorrhage and/or neurological defects when present in the central nervous system [13]. Sporadic (non-inherited) AVMs have recently been associated with activating mutations in genes related to the RAS/MAPK/ERK (rat sarcoma virus/mitogen activated protein kinase/extracellular signal-regulated kinase) signaling pathway [14] including *KRAS*, *MAP2K1*, and *BRAF*. The importance of this pathway in AVM formation is also demonstrated by the presence of inherited *RASA1* [15] and *EPHB4* [16] loss-of-function mutations in CM-AVM, which also result in dysregulation and activation of RAS/MAPK/ERK signaling. Patients with CM-AVM sometimes present with AVMs (about 30% of patients) and have several small capillary malformations (CM) and/or one large CM [17]. HHT, however, is highly associated with inherited loss-of-function mutations affecting TGF-β signaling [18], including *ENG*, *ACVRL1*, and *SMAD4*, which is involved in vessel stabilization and remodeling. HHT manifests with many variable symptoms, including nosebleeds, formation of telangiectasia (enlarged superficial blood vessels) in the skin and mucosa, and formation of AVM in the lungs, liver, and brain [19].

Slow-flow vascular malformations are grouped by the affected vessel type and include venous malformation (VM), capillary malformation (CM), lymphatic malformation (LM), and combined malformations [2]. CM typically appears at birth (often as port-wine stain birthmarks) or in early childhood, and are formed by dilation of capillary vessels, often within the dermis [20]. They can occur in syndromic (Sturge-Weber) or non-syndromic forms that are often associated with somatic activating mutations in the *GNAQ* gene [21]. LM, as the name suggests, are formed of abnormally enlarged or dysfunctional lymphatic vessels. Under the ISSVA classification, they include multiple subtypes including macrocystic and microcystic, as well as more complex disorders such as general lymphatic anomaly (GLA), Kaposiform lymphangiomatosis (KLA), and Gorham-Stout disease (GSD) [2]. These involve abnormal lymphatic cysts (macrocystic and microcystic), diffuse proliferative lesions (GLA and KLA), or progressive and bone-destructive lymphatic vessel overgrowth (GSD) [22]. Somatic activating mutations in the proto-oncogene *PIK3CA* have been identified in macrocystic and microcystic forms [23], implicating PI3K/AKT signaling in the pathogenesis of these conditions. However, mutations in the RAS/MAPK/ERK pathway have also been identified in cases of KLA (*NRAS* [24]) and GSD (*KRAS* [25]), implying that both pathways play important roles in lymphatic function and development.

A theme that emerges upon investigation of the genetic drivers of vascular anomalies is that mutations in some pathways are seen across multiple types of vascular anomalies, such as the presence of RAS mutations in angiosarcoma, sporadic AVM, and lymphatic malformations (see Table 1). This may, unsurprisingly, reflect the ubiquitous role of RAS/MAPK/ERK in growth, differentiation, and proliferation processes across cell types. However, other mutations/affected pathways do seem to be specific for a single vascular anomaly type. For example, TGF-β mutations (in *ENG*, *ACVRL*, and *SMAD4*) are specifically associated with AVM [18]. While a convenient explanation for this specificity would be that these genes are only expressed within the arterial compartment, this explanation is not supported by the literature. For example, SMAD4 expression is seen across all vessel types [26]. Therefore, the specificity of the mutations is more likely to be related to their specific function within a cell type, rather than specific expression. For example, a growing body of evidence suggests that SMAD4 is important for integrating normal cellular responses to fluid shear stress, which is higher in arterial cells [27]. Thus, it is thought that loss of this protein (via loss-of-function mutations) in cells experiencing high blood flow impairs flow-induced signaling, leading to a specific phenotype within these cell populations [28, 29].

Another interesting phenomenon is that different mutations in the same protein can contribute to the development of different types of vascular anomalies. For example, GNAQ p.R183Q mutations are found in Sturge-Weber syndrome [21], which is characterized by facial CM that affect the skin, eye, and nervous system. However, GNAQ p.Q209L mutations are most often found in congenital hemangioma [10], a type of vascular tumor. While the molecular and cellular effects of the different mutations are not yet fully understood, there is evidence that the p.Q209L mutation elicits a larger increase in pathway activation, compared to the p.R183Q mutation, thereby leading to different downstream changes to transcriptomic profiles [30]. While these discussions of SMAD4 and GNAQ mutations are merely small examples in the overall repertoire of vascular anomaly-causing mutations, they illustrate key concepts in how we are beginning to understand how these mutations function at the cellular level, which contributes to how they manifest at a phenotypic level.

### Venous malformation

Venous malformation (VM) is the most common subtype of vascular malformation. As a rare disorder, the incidence of VM is difficult to ascertain. Initial estimates yielded an incidence of 1–2 per 10,000 births [31] and more recent analyses have estimated a prevalence from 0.045% [32] to 0.06% [33]. They typically occur very early on in life, either being seen at birth or in early childhood, and can be somatic or, less frequently, inherited. However, many patients may not be diagnosed with a VM until later on in life, when symptoms worsen or become more noticeable. Often, puberty or pregnancy can spur increased growth of VM lesional vessels due to changes in the levels of sex hormones, which is when this disease may become more evident or require clinical appraisal [34]. Unlike benign vascular tumors, which can undergo spontaneous regression, VM never regresses and typically grows commensurately to the growth of the child [35]. Therefore, VM is a chronic condition that will be present throughout the course of an individual’s life.

VM often presents in patients as compressible blue or purple masses when located in the skin or subcutaneous tissue. Deeper lesions may be less evident and only present later in life when symptoms (such as pain) begin to develop [36]. They occur most often in the head and neck region (~ 40%) or in the extremities (~ 40%), and less frequently appear in the trunk or across multiple regions (~ 20%) [36]. An estimated 90% of patients present with unifocal disease, where they have only a single lesion [37]. Of the 10% that have multifocal disease (with multiple lesions), some have extensive sporadic disease while others have inherited forms, such as cutaneomucosal venous malformation (VMCM) and glomuvenous malformation (GVM) [37]. While there is little mortality associated with VM, morbidity for these patients can be high. They often experience severe and chronic pain, risk of bleeding, local swelling, and deformity of the affected tissue [36].

Blood flow is highly abnormal in VM lesional vessels and is often very slow or stagnant. This can contribute to abnormal coagulation and thrombosis, including the formation of phleboliths (thrombotic calcified masses) within the VM vessels which can contribute to chronic pain [38]. These processes can also progress to a more severe form of coagulopathy, known as localized intravascular coagulopathy (LIC). LIC is thought to occur due to the local activation of thrombin within the VM, which contributes to fibrin formation. This clinically manifests as elevation of fibrin degradation products, typically measured as D-dimer. It has been reported that up to 50–60% of VM patients will present with elevated D-dimer and this is associated with the presence of large or deep VM lesions [39]. However, it is important to note the differences between LIC that can occur with VM and the life-threatening coagulopathy (KMP) that can occur in locally aggressive vascular tumors. KMP is thought to involve platelet trapping in lesional vessels and therefore manifests with severe thrombocytopenia, a phenomenon that is not typically observed in VM [40].

Histology of VM lesions reveals the presence of abnormally structured and dilated veins [41]. These are lined by a single layer of EC and have abnormal coverage of mural cells, mainly vascular smooth muscle cells (vSMC). The muscle layer is discontinuous and while there is a lack of vSMC in some areas, it may be enlarged and disorganized in other vessel regions [42]. It has been postulated that the lack or disorganization of vSMC in VM may contribute to abnormal vessel dilation [43], however experimental evidence showing that restoring the recruitment of mural cells to the VM vessels would rescue the VM phenotype has never been published.

## Genetic causes of venous malformation

Increased understanding of the role that genetic mutations play in the pathogenesis of vascular anomalies has been essential for recent advances in how vascular anomalies are studied and treated. Seminal work from the 1990’s and early 2000’s identified the germline mutations responsible for many inherited forms of vascular anomalies, including the inherited forms of venous malformation [56]. Additional genetic studies in the 2000’s and 2010’s found many of the somatic mutations responsible for sporadic disease [56]. These discoveries have ushered in a new era in vascular anomaly research of genetic cellular and animal models, increased understanding of the cellular mechanisms that contribute to disease pathogenesis, and novel medical therapies, targeted to underlying disease mechanisms. In the following sections, we will specifically review the genetic discoveries made in patients with various types of VM, which are summarized in Table 2.

**Table 2:** Overview of venous malformation subtypes and genetics

Subtype	Inherited/sporadic	Clinical characteristics	Genes involved
Cutaneomucosal venous malformation (VMCM)	Inherited	Multifocal small VM lesions that most often appear in the skin and mucous membranes. New lesions can appear with age [47]	Inherited *TEK* (TIE2) activating mutations, most commonly p.R849W and p.Y897S/C [48–50]. May require a second-hit causing loss of the wild-type allele [42]
Glomuvenous malformation (GVM)	Inherited	Multiple VM lesions that are hard and painful with a cobblestone appearance. Contain unique undifferentiated mural cells (glomus cells) [46]	Inherited mutations that result in truncation of *GLMN* (glomulin) [46]
Familial cerebral cavernous malformation (CCM)	Inherited/sporadic	Venous lesions that appear in the central nervous system, consisting of dilated vessels with thin walls that can hemorrhage [45]	Inherited or sporadic loss-of-function mutations in *KRIT1* (CCM1), *CCM2* [54], and *PDCD10* (CCM3) [55]
Sporadic venous malformation (VM)	Sporadic	Unifocal (common) or multifocal (less common) lesions consisting of morphologically abnormal and enlarged veins [36]	Somatic activating *TEK* (TIE2) mutations [42, 64–66]. Most common mutation is p.L914F. Double *cis* mutations are also found (often p.Y897 with an additional substitution at p.R915 or S917) Also, somatic activating *PIK3CA* hotspot mutations, most often p.E542K and H1047R [51–53]
Blue rubber bleb nevus syndrome (BRBN)	Sporadic	Many (tens to hundreds) small VM lesions occurring in the skin or gastrointestinal tract, leading to bleeding [70]	Somatic double *cis* mutations in *TEK*, most common is p.T1105N-T1106P, as well as p.Y897-R915L/T1106 [71]

### Hereditary venous malformation and the discovery of TIE2 mutations

VMCM is a rare hereditary form of VM that was discovered to have an autosomal dominant pattern of inheritance that manifests with small, multifocal VM lesions [47]. It is known as “cutaneomucosal” VM because patients tend to accumulate many VM lesions throughout their skin and mucosal tissues. Genetic mapping studies of two affected families were the first to reveal a likely genetic culprit: a p.R849W substitution (c.C2545T) in the *TEK* gene, which encodes for the TIE2 (TEK receptor tyrosine kinase) protein [48]. Further studies identified this mutation and other *TEK* missense mutations (p.Y897S [49, 50], p.Y897C, p.R915H, p.R918C, p.V919L, p.A925S, and p.K1100N [50]) in additional families with VMCM. Studies of the mutant proteins in vitro revealed that mutations are universally hyperactivating, contributing to ligand-independent autophosphorylation of the receptor, although different mutations conferred varying degrees of activation [48–50]. In addition to germline genetic mutation, there is limited evidence that a somatic second-hit mutation, resulting in loss-of-function of the wild-type TIE2 allele, may be involved in lesion formation [42]. The idea of a two-hit mechanism was proposed by Knudson as occurring with inherited cancer-predisposition mutations [57]. This idea would provide biological rationale for the variable clinical presentation of VM lesions in these patients. Under this model, the germline mutation predisposes or “primes” EC to form VM lesions, however a somatic second-hit loss of the wild-type allele is necessary for lesion formation.

There are additional hereditary disorders, GVM and familial cerebral cavernous malformation (CCM), that can be associated with venous anomalies and therefore the ISSVA classification includes these within the venous malformation subcategory [1]. However, in clinical practice these are typically considered distinct entities from inherited and sporadic VM, based on clinical and histological characteristics and underlying genetic pathology. GVM lesions are distinguishable from VM lesions in that they are typically harder and more painful upon palpation, have a cobblestone appearance, and have unique glomus cells (thought to be vSMC that have undergone improper differentiation) that surround the abnormal venous channels [46]. GVM is therefore named for these distinctively rounded glomus cells that are pathognomonic for this type of venous anomaly. Familial mapping studies showed that causative mutations occur in the *GLMN* gene and universally result in premature termination of the glomulin protein, either via deletions, insertions, or point mutations [46].

Familial CCM is an autosomal dominant disorder which manifests as central nervous system lesions that consist of clusters of dilated and thin-walled venous caverns [45]. Germline, loss-of-function mutations have been identified in three primary genes, *KRIT1* (CCM1) [54], *CCM2* [54], and *PDCD10* (CCM3) [55]. Both familial and sporadic CCM can be caused by somatic second-hit mutations in the same genes [58]. Interestingly, studies investigating the genetics of sporadic and familial CCMs have discovered that many also harbor gain-of-function mutations in *PIK3CA* (whose role in VM is discussed in Sect. 2.3) [59, 60] and *MAP3K3* [61], implicating a multi-hit mechanism for the pathogenesis of this disease. Sporadic CCM is sometimes also associated with developmental venous anomalies (DVA), benign congenital malformations of brain veins, and these have also been found to harbor *PIK3CA* mutations in CCM patients [61]. Therefore, *PIK3CA* mutations may be causative for both conditions. While it is not entirely clear why these lesions are biased for the central nervous system (particularly the brain), evidence suggests that brain EC are uniquely dependent on the CCM pathway in maintenance of the blood–brain barrier and loss of CCM proteins disrupts the neurovascular unit [62] and impairs cell junction formation [63], contributing to abnormal vessel morphogenesis.

### Sporadic venous malformation and TIE2 mutations

Upon identification of germline genetic mutations as the causative agent for hereditary VM, it was postulated that somatic variants in the same genes could be responsible for sporadic forms of the disease. In a groundbreaking study of a cohort of sporadic VM patients, 50% were identified to have a somatic TIE2 mutation [42]. In the majority of these cases (86%), the mutation was a p.L914F substitution. Surprisingly, four patients had double TIE2 mutations in *cis* (on the same allele), all of which had a p.Y897 mutation (substituted for different amino acids: H, F, C, or S) with another substitution (p.R915C, R915L, or S917I) [42]. Additional studies have identified other less common mutations in TIE2, including missense mutations in the kinase and kinase insert domains and truncating C-terminal mutations [64–66]. It’s important to note that p.Y897C/S mutations have also been identified in the germline, where it has been proposed that somatic second-hit mutations resulting in loss of the wild-type allele may be required. Together, these observations would suggest that in some cases, these mutations are too weak to exert a clinically observable effect on their own and require a second-hit, either loss of the wild-type allele or gain of an additional somatic substitution on the same allele.

 While many different TIE2 mutations have been identified, large scale genetic studies of VM have clearly shown that p.L914F is the most common, occurring in 50–70% of TIE2-mutant sporadic VM cases and never in cases of inherited VM [42, 65–67]. While it was initially proposed to be only found in unifocal disease (which can be localized or extensive) [42, 68], recent evidence suggests that it can be found in some cases of multifocal disease [66, 69]. Furthermore, while the other TIE2 missense mutations are often found in double-hit scenarios (in which two TIE2 mutations occur in *cis*), p.L914F is almost always found on its own [65, 66]. However, Limaye and colleagues provided evidence that the ratio of cDNA to DNA of the TIE2 p.L914F mutant allele is amplified compared to the wild-type allele, in the range of 2–14 fold enrichment [42]. Possible mechanisms are somatic loss of the wild-type allele or selective transcriptional overexpression of the mutant allele, although the true cause is unknown. Regardless, these data may indicate that although the p.L914F mutation does not require a second-hit activating mutation in the same allele, it may require reduced amounts of competing wild-type TIE2 protein.

Blue rubber bleb nevus syndrome (BRBN) is an additional sporadic disorder involving TIE2 mutations and formation of venous anomalies. BRBN is clinically very distinct from other types of inherited or sporadic VM. Patients often develop many (tens to hundreds) small venous lesions throughout their body that are often cutaneous and can occur in the gastrointestinal tract, which is rarely seen with other disorders and is therefore pathognomonic for BRBN [70]. These gastrointestinal lesions are prone to frequent bleeding, increasing risk for the development of iron-deficiency anemia. Recently, somatic *cis* double-hit mutations were also found in the majority of a cohort of BRBN patients [71]. A third of these patients had the same double-hit (p.T1105N-T1106P) and the others had a p.Y897 mutation in combination with various second-hits (including p.R915L, R918, and T1106P). It should be noted that while the p.T1105N-T1106P double-hit mutation seems to be somewhat specific for BRBN, the other combination mutations follow a similar pattern as to those seen in sporadic multifocal VM, where a p.Y897 mutation is almost universal and can occur in combination with a range of additional second-hits [42].

It is important to notice that the difference between the clinical presentation of the two entities (BRBN and multifocal VM) may be due to differences in the timing of the mutations. The previously mentioned BRBN study noted that there was low level mosaicism present for one of the mutations in sporadic multifocal VM that was not present for the other mutation (one mutation was detectable in blood while the other was not) [71]. This implies that one mutation perhaps occurred earlier on in development, resulting in more widespread frequency of the mutation, which was then followed by a somatic (and limited) gain of the second mutation. However, the same was not found for the BRBN mutations. Here, they found that the p.T1105N-T1106P mutation was found at the same frequency and always on the same DNA read, implying a lack of cells with single mutations [71]. Strikingly, multiple lesions in different locations (but within a single patient) all had the same genetic alteration, despite evidence of germline inheritance or widespread mosaicism, which suggests either that the mutation occurred in an EC progenitor (capable of widespread dissemination) or that there is release and migration of mutant EC from lesions. Further work will be needed to determine which phenomenon is responsible.

### Sporadic venous malformation and PIK3CA mutation

While TIE2 mutations had been identified in the bulk of sporadic VM cases, there were still many patients whose genetic driver was not identified. Significant progress was made in this regard with the discovery of *PIK3CA* mutations in sporadic VM patients [51–53]. In these studies, they found *PIK3CA* mutations in 20–25% of patients and the majority of these mutations were “hotspot” mutations that are well-known drivers of various types of cancer (most often p.E542K and H1047R). *PIK3CA* encodes for the catalytic p110α subunit of phosphatidylinositol 3-kinase (PI3K) which is an important signaling node in the PI3K/AKT/mTOR pathway that has essential roles in cell growth, proliferation, and metabolism [72]. Furthermore, *PIK3CA* mutations were observed to be almost completely mutually exclusive with TIE2 mutations [51, 52, 65], suggesting that activation of downstream PI3K signaling is sufficient to cause formation of sporadic VM.

The effects of *PIK3CA* mutation and TIE2 mutation are not completely equivalent in the vasculature. This is apparent by the prevalence of both types of mutations across different types of vascular anomalies. Whereas TIE2 mutations are only found in cases of VM (including inherited and sporadic forms), *PIK3CA* mutations are often found in lymphatic malformation [23, 73] and in syndromic disorders that fall under the umbrella of *PIK3CA*-related overgrowth spectrum (PROS). PROS is thought to be caused by early but mosaic mutations in *PIK3CA* during embryonic development [74]. The higher frequency of the mutation (compared to somatic events) across a wider range of cell types and tissues results in a more severe and variable phenotype. Many patients experience tissue overgrowth affecting adipose, muscle, or skeletal tissue, and can also develop vascular malformations, including AVM, LM, CM, and VM (reviewed in [75]), which is expected considering the role of PI3K signaling in cancer. However, it is important to understand that the vascular anomalies associated with this syndrome do not undergo oncogenic transformation and are not malignant, in contrast to cancers that obtain *PIK3CA* mutations. Therefore, we can compare the vascular conditions resulting from germline/mosaic mutation of both *PIK3CA* (resulting in a range of vascular malformations, especially LM) and TIE2 (resulting in hereditary or multifocal VM). Whereas *PIK3CA* mutations are clearly capable of driving disease in both blood and lymphatic vessel types, thus far TIE2 mutation is specific for the development of VM.

### Mosaicism and allelic frequency

Based on this growing body of knowledge of the genetic mutations that drive formation of vascular anomalies, it is becoming clear that the timing or extent of the mutation during development plays an important role in the resulting phenotype. This is especially obvious in the case of *PIK3CA* mutations, where the effects of embryonic mosaicism result in severe and widespread phenotypic effects, due to the universal role of PI3K signaling across many cell types. However, the effect of TIE2 mutation in the mosaic state is perhaps not as striking, due to its specific effect on the venous vasculature. Nonetheless, from analyzing the frequency or presence of activating TIE2 mutations across various types of VM, we can attempt to draw several conclusions about the genetic nature of TIE2 mutation.

The first is that not all TIE2 mutations are equivalent. While there are several TIE2 mutations that are tolerated in the germline and cause hereditary disease, the most common somatic variant (p.L914F) has never been found in inherited VM cases. This would imply that the p.L914F mutation is embryonically lethal in the germline state or in cases of widespread mosaicism from an early developmental mutation event. This would also provide evidence as to why it may be necessary for a second-hit mutation, the loss of the wild-type allele, to occur for lesion formation in inherited VM. If the inherited mutation was strong enough to cause disease on its own, it would likely be lethal during development and would not persist. A similar phenomenon seems to occur in sporadic multifocal VM with *cis* TIE2 double mutations. Here, evidence has been found that one mutation is more widespread (mosaic) than the other [71], implying that it occurred earlier in development and likely without phenotypic effects. Only when a second-hit mutation occurs in the same allele will the combined effect on TIE2 be enough to drive VM formation.

In the case of TIE2 p.L914F, initial genetic studies of patient cohorts suggested that it may only be found in cases of unifocal disease, where it could implied that expression of the mutation is very limited (somatic/restricted mosaicism) [42, 68]. However, more recent reports have identified TIE2 p.L914F mutations in some patients with multifocal disease [66], including a form of sporadic VM known as Bockenheimer disease, which is characterized by extensive VM lesions that are typically found throughout an entire limb and that extend into all tissue layers [69]. The existence of TIE2 p.L914F in these diseases implies that widespread mosaicism of the mutation is possible, although the extent of embryonic mosaicism is likely very limited by the deleterious effects of the mutation.

Studies of TIE2 mutation mosaicism in sporadic VM disease is complicated by the fact that the variant allele frequency (VAF, the proportion of sequences with a genetic variant at a specific DNA locus) is typically very low in clinical VM samples. In analyses of patient cohorts, the VAF of TIE2 p.L914F is often less than 10% [76, 77]. The cause of the low VAF is not entirely clear and may be due to several factors. First, the mutation is often thought to be restricted to lesional EC [78] and the tissue samples taken upon biopsy and surgical removal will often include relatively large proportions of surrounding tissue and blood, which may effectively dilute out signal from lesional cells. Furthermore, it is unknown if VM lesions consist of only mutant EC, or if wild-type EC are also recruited to the lesion, although recent experimental evidence would suggest that wild-type EC are repelled from lesional vessels [79]. Finally, genetic mosaicism within the analyzed tissue would contribute to changes in VAF. Thus, more detailed genetic analyses of patients and further experimentation with VM animal models are needed to decipher how allelic frequency and mosaicism play a role in the phenotypic manifestation of VM-causative TIE2 mutations.

Beyond VM, mosaicism has been observed in other types of vascular anomalies. CCM (discussed in Sect. 2.1) provides a particularly good example, as several groundbreaking clinical and bench research studies have generated key insights into CCM pathogenesis. In familial cases of CCM, evidence suggests that a two-hit mechanism exists, in which all cells have loss of one CCM gene allele (due to germline inheritance) and the second allele is then somatically lost within lesional cells, resulting in genetic mosaicism [80]. Further complicating the genetic makeup of these lesions, additional somatic gain-of-function mutations in *PIK3CA* and *MAP3K3* have been identified in lesional tissues, typically at low VAF (less than 10%) [54]. Currently, limitations in molecular techniques (and the scarcity of tissue samples from CCM patient lesions) make it difficult to determine exactly in which order mutations appear across time and where cells with a specific mutational profile are located in the CCM lesion. However, these research endeavors are beginning to show that the appearance of somatic mutations over time can result in different cell populations within normal and lesional tissue. Finally, the role of these cell populations in lesion growth is complicated by the ability of mutant EC to undergo clonal expansion and also to recruit or repel genetically wild-type cells. Mouse models of CCM seem to indicate phases of both processes, in which CCM-mutant cells first undergo clonal proliferation to form a CCM lesion and later recruit wild-type cells to sustain lesion expansion [81].

## Treatment modalities for venous malformation

Current treatment options for VM patients are limited. Sclerotherapy, and sometimes surgery, are first-line treatments for patients for whom these options are possible, but these methods are invasive, can require several treatments, have potential complications and side effects, and are not always curative. For those patients whose VM lesions are not eligible for sclerotherapy/surgery, options are typically limited to symptom-modulating therapies. New discoveries on the genetic causes and cellular mechanisms that contribute to VM pathogenesis have paved the way for crucial advancements in targeted medical therapies that will provide much-needed options for patients not eligible for, or that have failed, traditional treatments.

### Diagnosis

Effective treatment for a VM patient begins with correct differential diagnosis. Clinical presentation is often the first step, as VM lesions (especially those that are superficial) often have readily distinguishable characteristics, including blue/purple color, the ability to be compressed, and enlargement upon increased venous pressure, such as with a Valsalva maneuver [40]. Upon suspicion of a VM, imaging is often the next step in a definitive diagnosis. Duplex ultrasonography (which combines the structural imaging properties of standard ultrasound with the blood flow imaging capabilities of Doppler ultrasound) is often the first choice, due to its availability, non-invasiveness, low cost, and low risk of radiation exposure. VM vessels often appear as well-marginated masses with ectatic venous spaces upon ultrasound [82]. Furthermore, Doppler analysis will show very slow or no flow, which is useful when distinguishing from fast-flow lesions [82]. In a subset of patients, phleboliths may also be seen within lesions, which is highly indicative of a VM. Magnetic resonance imaging (MRI), and less often computed tomography (CT), are sometimes used when the diagnosis is unclear with ultrasound, such as with deep and inaccessible lesions. These methods can provide more definitive imaging of the lesions and, when combined with angiography, can map out feeding and draining vessels from the lesion and determine lesional flow dynamics [83].

### Sclerotherapy and surgery

For many patients, sclerotherapy is a first-line option in the treatment of their VM. The founding principle of sclerotherapy is that percutaneous injection of a damaging agent (the sclerosant) into the lesional vessel will injure the abnormal endothelium, causing closure or shrinkage of the lesion [84]. Sclerosing agents fall into several categories, include alcohols (often ethanol), detergents (including sodium tetradecyl sulfate (STS)), and chemotherapeutics (often bleomycin) [85]. Choice of a sclerotherapy agent is a double-edged sword; more damaging agents are more likely to result in positive lesional response but are also more likely to cause complications [85]. Illustrating this, ethanol and STS are two very common agents used in the treatment of VM. Ethanol has generally been found to have the highest success rates, but also the highest risk of complications. On the other hand, STS has moderate success rates, but lower rates of complications. These complications include severe swelling and pain, local tissue damage and necrosis, and disseminated intravascular coagulopathy [85]. Furthermore, patients often require repeated treatments (especially for extensive lesions) and some studies show that over 50% of patients experience full or partial recanalization of the lesion [86].

Surgery is sometimes an option for patients, either after initial sclerotherapy treatment or as first-line. Clinical factors that can complicate resection are the risks of bleeding, location (such as deep lesions or those next to critical structures), and if the lesion is localized or extensive [87]. While surgery can be curative for some patients, many still experience recurrence of lesion growth and symptoms, which can require additional treatments [88]. Therefore, while sclerotherapy and surgery can be successful for a cohort of VM patients, many patients are either not eligible for these treatments or experience recurrence. This can be especially challenging when these options are extremely invasive and require repeated hospital visits, imaging, and interventions. Thus, there is a critical need for new therapies that are less invasive and more effective, especially for the more vulnerable pediatric population that will have these lesions throughout their life.

### Targeted medical therapies

With the discoveries of the genetic drivers and molecular mechanisms that drive VM formation, the new therapeutic frontier in treatment of VM is the identification of targeted medical therapies. Even before genetic testing of patients was common, the known role of PI3K/AKT/mTOR signaling in vascular development and angiogenesis provided the rationale for the use of rapamycin (also known as sirolimus), in the treatment of patients with severe vascular anomalies [89]. Rapamycin is a drug that primarily inhibits mTOR (mammalian target of rapamycin) complex 1 (mTORC1, which is highly involved in cell growth), but also inhibits mTORC2 (involved in pro-survival signaling through AKT) when given chronically [90]. Results from the first trial of rapamycin in patients with complex vascular anomalies demonstrated at least partial response in over 80% of patients, with impressive results seen in patients with lymphatic anomalies and KMP [89].

However, rapamycin was not used for VM patients until investigation of the molecular consequences of mutant TIE2 in experimental models led to the discovery of increased signaling and dependence on PI3K/AKT/mTOR signaling [43, 91, 92]. This pathway has been successfully targeted in VM experimental models with rapamycin [51, 92–95]. Boscolo and colleagues (2015) performed the first clinical trial of rapamycin treatment in six patients with VM and found that patients experienced increased quality of life (including reductions in lesional pain) and had small reductions in lesion size [92]. This was followed by a 2018 trial in another small set of VM patients where similar results were seen [96]. The PERFORMUS trail (published in 2021) was the first to look at rapamycin in a much larger cohort of patients, where they observed that VM patients did not experience significant decreases to lesion size but did have positive benefits to quality of life [97]. Patients on rapamycin did experience common adverse events, including ulcers, upper respiratory infections, and headaches, and some more serious adverse events, including sepsis and infection [97]. More recently, a meta-analysis of multiple trials in which rapamycin was used in VM patients reaffirmed these results, in which it is moderately effective in reducing lesion size but is very often beneficial for improving quality of life, reducing pain, and decreasing D-dimer levels [98]. All of these results have contributed to the off-label use of rapamycin in VM patients, although FDA approval still has not been achieved.

Due to the variable effects of rapamycin in patients, there is great interest in investigating other inhibitors of PI3K/AKT/mTOR signaling. One of the most promising options is alpelisib, a PI3K inhibitor that is approved for treatment of *PIK3CA*-mutant breast cancer [99]. Two recent trials have tested alpelisib in *TEK*- and *PIK3CA*-mutant VM, with promising results [100, 101]. They observed moderate reductions in lesion size (21% for *TEK* mutant cases and 53% for *PIK3CA* mutant cases) but universal improvements in D-dimer and quality of life. However, the studies did note minor side effects (such as headaches and nausea) as well as more severe instances of hyperglycemia, mucositis, and abdominal pain. It is important to note that these studies were able to use doses lower than those used in adult cancer patients, which may indicate that these patients have a wider therapeutic window, meaning that they may be able to take lower, more tolerable doses and still achieve therapeutic effect. More trials will be needed to investigate the long-term consequences of alpelisib in VM patients. It is also interesting to note the disparity in lesion reduction in the *TEK* mutant cases compared to *PIK3CA.* Further experimental work is needed to determine if mutation status is associated with distinct clinical responses to PI3K/AKT/mTOR modulating drugs.

## Model systems of venous malformation

The development of model systems that allow for the investigation of how *TEK* and *PIK3CA* mutations affect endothelial signaling and cellular phenotypes have been essential for increasing our understanding of how these mutations cause VM. Just as importantly, they allow for the preclinical testing of drug candidates, which has paved the way for the development of new medical therapies for VM patients.

### In vitro systems–endothelial cell lines and 3D modeling

The first systems used to elucidate the cellular and molecular consequences of VM-causative mutations were primary human EC cell lines. Human umbilical vein endothelial cells (HUVEC) are typically the cell line of choice for vascular biology studies. These are isolated from umbilical veins soon after birth and yield pure cultures of EC that replicate for many passages in vitro [102]. Researchers then use viral overexpression systems to introduce the desired mutant gene into the host cell genome. Upon the discovery of TIE2 p.L914F in sporadic VM, transduced HUVEC were used to study downstream signaling and protein localization [42]. Further studies in HUVEC expanded on these results, investigating the less commonly found VM-causative TIE2 mutations and identifying specific cellular signaling pathways and phenotypes associated with various mutations [43, 91]. Similar experiments have been performed with *PIK3CA*-mutant HUVEC [103]. It is important to note that these models depend on viral overexpression of the mutant gene, so these experiments always utilize a control HUVEC line that overexpresses the wild-type protein. This problem can be addressed by using patient-derived EC or with gene-editing of stem cells, discussed in later paragraphs.

These cells were also used in 3-dimensional (3D) assays that allow for the investigation of angiogenic behavior in more relevant environmental contexts. Nätynki and colleagues showed that, when embedded in fibrin gels, spheroids formed of TIE2-mutant cells showed decreased sprouting activity and increased ability to form hollow, cystic structures [91]. Cai and colleagues developed a lumen formation assay with TIE2 p.L914F-mutant HUVEC, in which cells are coated on carrier beads and then embedded within a fibrin gel with supporting fibroblasts cultured on top [94]. Here, they found that wild-type HUVEC sprout from the beads and form thin, lumenized structures that resemble typical vessels. However, TIE2 p.L914F mutant EC form dilated cysts, reminiscent of VM vessels found in patients [94]. Importantly, they were also able to extend this model to investigate the effects of mutant EC on mural cell recruitment, a first step into understanding why mural cells are often lost or disorganized in VM lesions. New advances in 3D modeling have been used in recent studies that seek to improve the physiological relevance of these systems. Many new systems use microfluidic units that flow cell culture media over cells cultured in monolayer or 3D tubes, mimicking blood flow and providing flexible environments for EC structures to grow [103, 104]. These models will be especially important in determining how altered blood flow dynamics may contribute to VM phenotypes, as it is becoming increasingly appreciated that EC response to flow plays an essential role in vascular development and function.

In addition to engineered EC cell lines, patient-derived VM cell lines have also proven to be a useful tool in the study of VM. The isolation of patient-derived EC was first reported in 2018, where immunomagnetic beads for CD31 (a surface marker specific for EC) were used to isolated EC and non-EC populations both from digested VM tissues and from lesional sclerotherapy blood [78]. Using these cells, they were able to show that somatic VM-causative *TEK* and *PIK3CA* mutations were only found within the EC population and that patient-derived EC had upregulation of signaling pathways previously investigated using engineered cells. Patient-derived VM cell lines have further been used in studies of ABL (Abelson kinase) and Semaphorin signaling in TIE2 mutant EC [79, 95]. One important limitation to note about the use of patient-derived cell lines is that isogenic control cell lines have not yet been available. Normal (non-mutant) primary EC from human tissues are ethically not available and can be difficult to expand in culture, so alternative controls (such as HUVEC) are often used instead [78]. Future studies may address this issue by using gene-editing to derive isogenic *TEK* or *PIK3CA* wild-type lines from the VM-mutant lines, but these experiments have not yet been recorded in the literature.

Finally, the most recent advance in cell culture modeling of VM EC has been the generation of protocols to derive EC from iPSC (inducible pluripotent stem cells). It has been well-established in the vascular biology field that direct gene-editing of primary human EC (including HUVEC) is rarely feasible, due to difficulty transfecting these cells, their limited lifespan in vitro, and their inability to generate single-cell clonal populations [105]. However, iPSC lines offer solutions to these problems; they are more easily expanded from single cells to make clonal populations, they are often easier to genetically manipulate with CRISPR, and with the proper protocols, they can be differentiated into many different cell types [106]. Initial protocols to convert iPSC to EC relied on chemical stimulation with differentiation factors including BMP4, GSK3 inhibitor, and VEGF [107]. More refined protocols now also use forced expression of the ETV2 (E26 transformation-specific variant 2) transcription factor, which plays a role in differentiation of EC during development, to more efficiently drive cells to the EC lineage [108, 109]. Two groups have since used variations of these protocols to generate CRISPR-edited EC lines expressing TIE2 p.L914F [104, 107], which have been used to study changes in gene expression, cellular phenotypes, and ability to form blood vessels in xenografts.

### In vivo systems–xenograft mouse models

An important step in increasing the physiological relevance of VM experimental models was the addition of an in vivo component: murine xenografts. The first two iterations of this technique in the study of TIE2-driven VM were both published in the same month in 2015 [91, 92]. Boscolo and colleagues used HUVEC overexpressing either wild-type TIE2 or mutant TIE2 p.L914F which were suspended in Matrigel and injected subcutaneously into immunodeficient nude mice [92]. Over a few weeks, the Matrigel plugs containing TIE2-mutant EC become vascularized, as the human EC connected to the host murine blood vasculature, enabling perfusion of the new vessels. Furthermore, the blood vessels that form from TIE2 p.L914F HUVEC are massive with dilated lumens. In contrast, the plugs containing wild-type EC fail to vascularize and functional vessels are rarely seen. The plugs can be monitored longitudinally over time by using calipers to measure the plugs on live mice. Upon resection, plug weight can also be used as a proxy for vessel growth in the xenograft and it can be fixed, sectioned, and stained to reveal the histology of the xenograft vessels. This model was important for preclinical testing of rapamycin, which led to its first use in a small cohort of VM patients [92]. The second xenograft model, published by Nätynki and colleagues, was performed with some differences in technique [91]. Here, they first formed spheroids from TIE2 wild-type or TIE2-mutant HUVEC (several types of TIE2 mutations were tested, including hereditary, sporadic, and double-mutant). These were then suspended in mixed Matrigel solutions that were injected subcutaneously in immunodeficient SCID mice. After 10 days, plugs with TIE2-mutant cells also became vascularized, with abnormally dilated vessels [91]. However, this model was not used to test any potential targeted therapy.

The xenograft model generated by Boscolo and colleagues was further expanded on with the use of patient-derived VM EC cell lines, which recapitulated the formation of abnormally enlarged vessels within the xenograft plugs [78, 110]. It has also since been used to test ponatinib (an ABL kinase inhibitor) in the treatment of VM lesions [95] and to investigate the role of chemorepellent Semaphorin proteins in the interaction of wild-type and TIE2-mutant EC during the formation of VM lesions [79]. These studies show the utility of this model in studying the aberrant signaling processes that drive VM lesion formation and the therapies that can be used to target them. They also show that the xenograft systems can be used to study cell interactions by mixing relevant cell types prior to injection.

### In vivo systems–genetically engineered zebrafish and mouse models

While in vitro and xenograft models have yielded crucial information in the study of VM pathogenesis, the gold standard for investigation of developmental diseases are genetic animal models. These more closely recapitulate the pathological processes that would occur in patients, where a genetic mutation is introduced during physiological development with resulting effects on bona fide blood vessels.

A commonly used animal model in vascular biology is the zebrafish, which is relatively easy to genetically modify, is high-throughput, and the transparency of embryos allows for non-invasive imaging of vascular structures. In the study of VM, Du and colleagues generated a transgenic zebrafish line with the TIE2 p.R849W mutation, which developed defects in the caudal vein plexus and other structures [111]. Later, Bell and colleagues also used zebrafish to study two *TEK* variants of unknown significance that were identified in VM patients, as well as other known *TEK* mutations (including p.L914F and p.T1105N-T1106P) [112]. They found that these mutations again caused malformations of the caudal vein plexus, mimicking VM, which were partially responsive to rapamycin treatment. Finally, Brunsdon and colleagues reported (in a preprint at the time of this review) that zebrafish expressing *PIK3CA* mutations developed vascular malformations [113]. In total, these articles show that progress is being made in the development of non-mammalian models that contain VM causative mutations and develop VM-like phenotypes. The benefits of these zebrafish models is that they are more efficient to generate, allowing several mutations to be studied in parallel, vascular phenotypes can be observed visually or with whole-mount fluorescent methods, and the effects of therapeutics can be determined efficiently. However, the anatomical structures and systems of zebrafish are not as closely related to those in humans as in mammalian model systems, reducing the translatability of these findings.

Therefore, mouse models are also commonly used in the study of vascular anomalies. However, because the mutations that cause VM are often not tolerated in the germline, inducible systems are needed, where expression of the mutant gene does not occur until an additional stimulus is provided. This is typically achieved in mice with the Cre-lox system, which requires, within the same cell, expression of Cre recombinase from one genetic construct and engineering of a second genetic construct that is controlled by flanking *loxP* (floxed) sites. The first genetic animal models of VM were achieved with Cre-inducible *PIK3CA*^*H1047R*^-mutant mice [51, 52]. In both publications, they found somewhat unexpectedly that when they crossed *PIK3CA*-mutant mice with *Sprr2f-Cre* mice [52] (thought to be specific for uterine epithelial cells but can be expressed in EC) or *T-CreER*^*T2*^ mice [51] (expressed in embryonic mesoderm), mice developed spinal or cutaneous vascular malformations, respectively. Further investigation revealed these lesions to be histologically similar to patient VM lesions, validating these as the first genetic models of *PIK3CA*-driven VM. More recent studies have built upon these by using EC-specific Cre drivers (including *Pdgfb-CreER* and *Cdh5-CreER*^*T2*^). The first study was performed by Di Blasio and colleagues (2018) which showed that, upon local tamoxifen induction in the leg muscle, *Cdh5-CreER*^*T2*^; *PIK3CA*^*H1047R*^ mutant mice developed vascular lesions with massively enlarged blood vessels [114]. In Kobialka and colleagues (2022), they used *Pdgfb-CreER* to drive expression of *PIK3CA*^*H1047R*^ during various stages of postnatal mouse development and then measured vascular changes in the mouse retina [115]. They found vascular lesions (areas of abnormal vascular overgrowth) within the retinas of mutant mice. Furthermore, they reported that lesion formation occurred during stages of active retinal angiogenesis, that mutant EC have higher rates of proliferation, and that miransertib (an AKT inhibitor) prevented formation of lesions [115].

Further work used various Cre drivers to look at the effects of *PIK3CA*^*H1047R*^ mutation during embryonic development in only lymphatic EC (*Vegfr3-CreER*^*T2*^), only blood EC (*Vegfr1-CreER*^*T2*^), or all EC (*Cdh5-CreER*^*T2*^) [116]. Expression in all EC resulted in both lymphatic and blood lesions, expression in only lymphatic EC resulted only in lymphatic lesions, and expression only in blood EC resulted in blood lesions. While expected, these results were important for confirming the dual role of *PIK3CA* mutation in both LM and VM and providing evidence that the cell type-of-origin plays important roles in the resulting phenotype [116]. The same lab built upon the *Vegfr1-CreER*^*T2*^; *PIK3CA*^*H1047R*^ model by locally inducing Cre expression via topical administration of 4-OHT to the ears of 3-week old postnatal mice [117]. These mice developed blood-specific vascular lesions within the ear skin that they showed had clonal expansion of mutant EC and a dependency on TIE2 signaling [117].

More recently, Bischoff and colleagues generated genetic animal models of mutant TIE2 p.L914F that recapitulate VM phenotypes. Upon generating a transgenic murine line that expresses TIE2 p.L914F under the control of a Cre-inducible construct, we first examined the phenotypic effects of the mutation during mouse development [118]. By crossing *TIE2*^*L914F*^ mice with the constitutive EC-specific *TIE2-Cre* driver line, we were able to determine that early developmental expression of the mutant gene caused embryonic lethality and impaired vascular remodeling [118]. These data are in line with observations made in VM patients, where the TIE2 p.L914F mutation is never found in the germline state due to its supposed developmentally lethal effects. Furthermore, we have more recently reported that we can drive mosaic embryonic expression of mutant TIE2 by utilizing the *CMV-Cre* driver line [119]. Here, mosaic expression of TIE2 is partially embryonically lethal, but surviving mutant mice live into adulthood and ubiquitously develop a VM-like vascular phenotype, with abnormally enlarged veins.

## Overview of TIE2 signaling

**Fig. 1:**
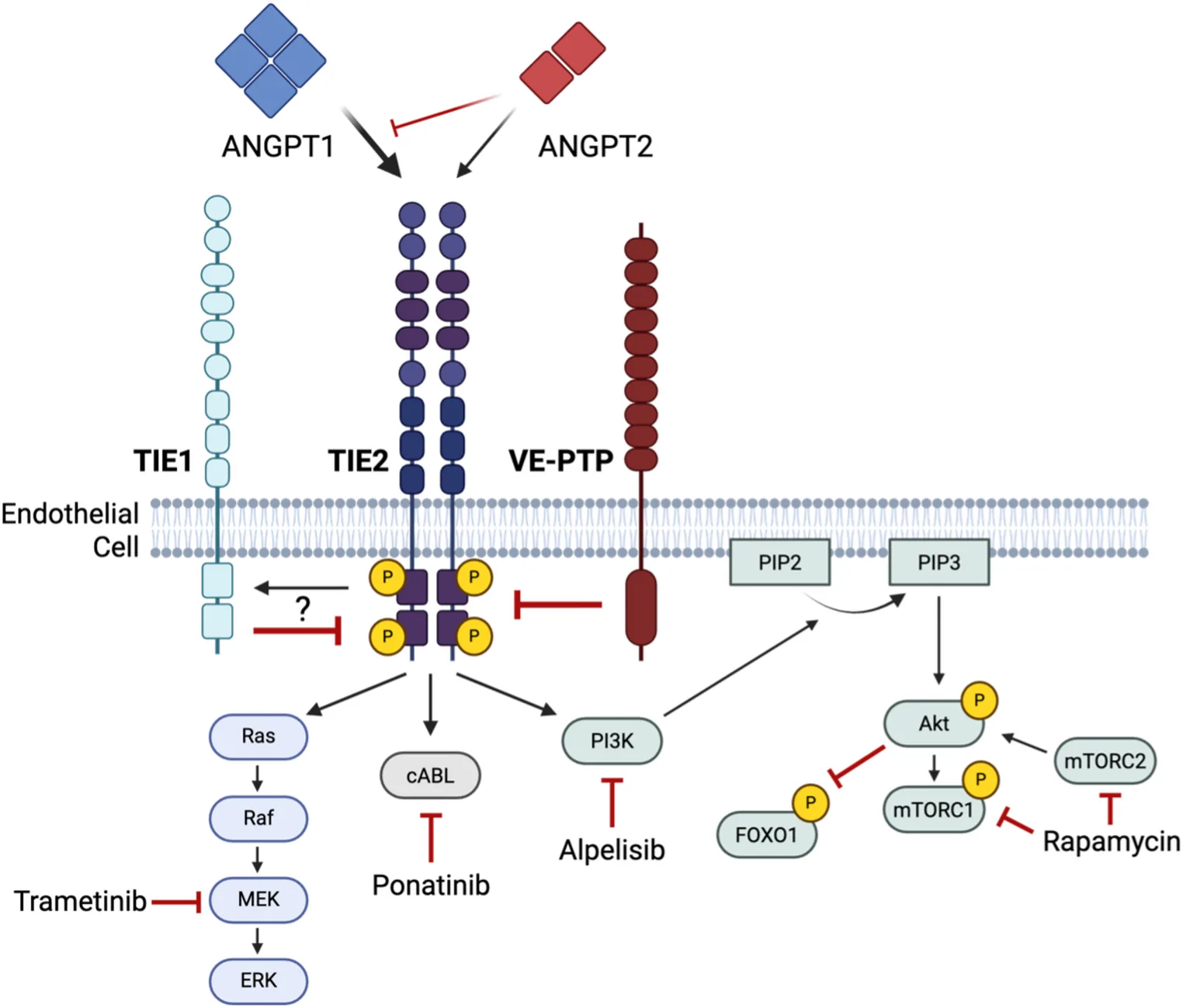
Summary of the TIE2 signaling pathway, modulators, and targeted therapeutics. Created with Biorender.com

As the ability of mutated TIE2 to drive vascular disease would suggest, TIE2 signaling is important for normal EC function, which contributes to vascular development and vessel stability. However, TIE2 signaling must remain balanced, as there are consequences in cases of both too much and too little activity. Relatedly, TIE2 signaling is acted on by several opposing forces which function to maintain TIE2 signaling during vessel quiescence or modulate signaling when certain environmental stimuli are present. Therefore, an understanding of how TIE2 functions at the molecular level is essential for investigation of how signaling goes awry in formation of VM. A summary of the TIE2 signaling components discussed in this section is provided in Fig. 1.

### TIE2 receptor expression and structure

Early investigations of TIE2 expression suggested that it is predominantly expressed by EC [120, 121]. However, additional work has shown that TIE2 is expressed in early progenitor cells during embryogenesis that contribute to both endothelial and hematopoietic lineages and it functions in early hematopoiesis [122, 123]. While TIE2 expression is lost or is very low in differentiated hematopoietic cells, it has been shown to be expressed on some hematopoietic stem cells in the adult bone marrow [124], as well as some monocytes [125]. TIE2 is a type 1 transmembrane (single-pass) receptor tyrosine kinase (RTK) and, like other RTKs, its activation is dependent on receptor dimerization or oligomerization with autophosphorylation. Its extracellular portion, which is responsible for ligand binding, consists of three immunoglobulin (Ig) domains, three epidermal growth factor (EGF) repeats, and three fibronectin (FN) type III repeats [126]. Mapping experiments suggest that ligand binding is achieved by the first Ig domain in combination with the EGF repeats [127]. Homodimerization between TIE2 receptors is mediated by the membrane-proximal fibronectin 3 (FN3) domain [127]. Studies of the structural consequences of ligand binding also suggest that dimerization is insufficient to cause receptor activation and higher-order oligomeric receptor clustering is thought to be needed [126, 128].

The intracellular portion of the receptor (residues 808–1124) contains the kinase catalytic function and its crystal structure was discovered by Shewchuk and colleagues (2000) [129]. It contains an N-terminal kinase domain (824–915) and a C-terminal kinase domain (937–1096). Like other protein kinases, it contains an activation loop which regulates kinase activity and in turn is regulated by phosphorylation of residues within itself. The TIE2 activation loop is composed of residues 982–1008 and includes Y992, whose phosphorylation is often used as an indication of TIE2 activation. The nucleotide binding loop, which is responsible for binding ATP during catalysis, is located at residues 831–836 and takes on a self-inhibitory conformation that blocks the ATP binding site. The TIE2 C-terminal tail (1096–1124) contains tyrosine sites that have been shown to bind SH2 and PTB domains (both implicated in phosphotyrosine binding) on other proteins. In addition, it seems to play a unique role in TIE2 in that it is positioned to block the substrate binding site, therefore acting to auto-inhibit the protein [129]. This may provide an explanation as to why some VM-causative mutations are located within this domain, as they may interfere with this inhibitory activity. Furthermore, all VM-associated TIE2 mutations are found in the kinase domains or in the C-terminal tail, indicating that deregulation of the kinase activity is essential for pathogenesis. However, the structural consequences of these mutations have not yet been elucidated.

### Angiopoietin-1 and -2

The angiopoietin family of proteins are ligands for TIE receptors. There are several members of this family, numbered as Angiopoietin (Angpt) 1–4, although regulation of TIE2 signaling mainly comes from Angpt1 and Angpt2 [130]. Angpt1 is a TIE2 agonist, while Angpt2 has been found to act as both agonist and antagonist in different contexts [131]. On its own, Angpt2 can increase TIE2 phosphorylation, PI3K pathway activation, and drive angiogenic phenotypes in functional assays, although less potently than Angpt1. However, the addition of Angpt2 and Angpt1 together results in a reduction of signaling compared to Angpt1 alone [131]. Structural analysis of Angpt/TIE2 binding has yielded clues as to how these ligands function to activate or inhibit TIE2 signaling. The general domain structures are similar for Angpt1 and Angpt2. In their monomeric form, they contain three domains: an N-terminal domain thought to be responsible for multimerization, followed by a coiled-coil (C) domain thought to be responsible for dimerization, and finally a C-terminal fibrinogen-like (F) domain that is responsible for binding TIE receptors [132].

Importantly, the multimerization of Angpt ligands is essential for their ability to activate TIE2. Electron microscopy has shown that Angpt1 exists in tetrameric, hexameric, and even higher order structures. Functional analyses further showed that tetramerization was minimally required for TIE2 activation [132]. When attempting to engineer activating variants of Angpt1, multimeric forms are much more effective at stimulating TIE2 activation in vitro [133]. In addition, unlike some other types of RTK, TIE2 doesn’t appear to undergo significant conformational change upon ligand binding, implicating ligand-induced clustering as a primary mechanism of activation [134]. Furthermore, Angpt1 and Angpt2 appear to bind TIE2 in the same way, raising questions as to how they can elicit different levels of activation. One hypothesis is that Angpt2 may natively exist in lower-order structures (i.e. dimers rather than tetramers), which are less efficient at TIE2 clustering and activation [134]. Other evidence indicates that small changes in the amino acid sequence of Angpt2 cause it to be less effective at disrupting TIE2/TIE1 heterodimers, which can be inhibitory in some contexts [134, 135]. An additional layer of regulation occurs with the relative amount of Angpt1 and Angpt2. Angpt2 has been shown to be a weak agonist of TIE2 in the absence of Angpt1 and an antagonist of Angpt1-induced activation [136]. This implies that in conditions of higher TIE2 activation from Angpt1, addition of Angpt2 appears antagonistic, as it competes for TIE2 binding sites.

### Regulation by TIE1 and VE-PTP

TIE2 has a highly related family member known as TIE1 (encoded by the *TIE1* gene), which is considered an orphan receptor and does not directly bind to Angpt ligands on its own [137, 138]. While TIE1 has been shown to have functional kinase activity [139], its lack of a known ligand makes it unclear how it functions in vivo. However, it does have known roles in modulation of TIE2 signaling by angiopoietins. First, in vitro evidence suggests that the TIE2 ligand Angpt1 can activate TIE1, but only in the presence of TIE2, perhaps due to phosphorylation of TIE1 by activated TIE2 [140]. Furthermore, the presence of TIE1 seems to blunt activation of pathways downstream of TIE2 signaling [140]. However, conflicting reports suggest that TIE1 is necessary for baseline TIE2 activation in vivo [141]. While these reports have not been fully reconciled, it has become apparent with new research that the effects of TIE1 on TIE2 signaling are context dependent. For example, TIE1 seems to differentially regulate TIE2 during different processes; it negatively regulates TIE2 signaling during sprouting angiogenesis but positively regulates it during vascular remodeling [142]. Furthermore, it has been shown that inflammatory processes can drive proteolysis and shedding of soluble TIE1 ectodomain, which interferes with TIE2 activation [141].

While it would be useful to determine whether signaling effectors downstream of TIE1 and TIE2 are different, investigation of these pathways are complicated by the fact that both receptors are often co-expressed in EC and that the primary effect of TIE1 is in regulating TIE2 signaling. Initial experiments in artificial cellular systems that lack TIE2 suggested that TIE1 is capable of binding and activating PI3K [139]. In addition, modulation of TIE1 expression in cancer cells also seemed to directly impact PI3K activation [143], which may indicate that TIE1 shares this downstream effector with TIE2. More recently, reports have yielded additional clues on the in vivo role of TIE1 signaling. It was shown that TIE1 expression precedes TIE2 expression in lymphatic precursor cells, TIE1 kinase activity is essential for early lymphatic development (whereas TIE2 is not), and it likely signals through FOXO1 in this context [144]. The important role of TIE1 in lymphatic vessels is further illustrated by the discovery of loss-of-function TIE1 mutations in patients with primary lymphedema [145]. Biochemical and cellular studies of these TIE1 mutants in cells expressing low amounts of TIE2 again suggest that TIE1 does similarly signal through PI3K [145]. However, results in cardiac EC indicate that only knockdown of TIE2 (and not TIE1) significantly impacts EC function, indicating in this context that the role of TIE1 is as a modulator of TIE2 activity and does not signal independently [146]. In total, these results indicate that TIE1/TIE2 signaling is complex and context dependent. Future studies seeking to delineate the independent roles of both receptors should be sure to consider the cellular context of the experiments and the relative expression levels of TIE1 and TIE2.

Further illustrating the complexity of balanced TIE2 signaling is the role of VE-PTP (vascular endothelial protein-tyrosine phosphatase), which is specifically expressed in vascular ECs and has been shown to de-phosphorylate (inactivate) TIE2 [147]. It has important roles in regulating TIE2 signaling and is therefore required for blood vessel development [147]. Its ability to downregulate TIE2 activation is demonstrated by studies showing that VE-PTP sequestration in vivo is associated with increased TIE2 phosphoryation [148] and that administration of VE-PTP inhibiting antibodies can increase TIE2 activation and signaling [149]. The expression of VE-PTP may also be an important contextual consideration in the differential effects of Angpt2 signaling. A study by Souma and colleagues (2018) suggests that VE-PTP expression is absent in the lymphatic vasculature, which makes TIE2 more susceptible to activation by Angpt2. In the blood vasculature VE-PTP is more highly expressed, and can modulate TIE2 activation and promote antagonism by Angpt2 [150].

### PI3K/AKT/mTOR signaling

PI3K/AKT/mTOR signaling is an essential and conserved pathway in eukaryotic cells that plays a role in promoting cell phenotypes including survival, growth, and metabolism [72]. Simplistically, signaling is often initiated by external stimuli recognized by RTKs, that first activate PI3K to prompt downstream signaling [151]. PI3K consists of two subunits: the catalytic p110 subunit (which includes p110α, the product of *PIK3CA*) and the regulatory p85 subunit. Activation of PI3K by RTKs is achieved by recruitment of the p85 subunit to phospho-tyrosine residues on an activated RTK, effectively relieving inhibition of the catalytic subunit and recruiting the PI3K complex to the lipid membrane. PI3K is a lipid kinase and it converts PIP2 (phosphatidylinositol 4,5-bisphosphate) to PIP3 (phosphatidylinositol (3,4,5)-trisphosphate) [152]. PIP3 acts as a second messenger in this system and functions in recruiting AKT (protein kinase B or PKB) to the membrane, where it undergoes phosphorylation and activation by PDK1 (3-phosphoinositide-dependent kinase 1) on threonine 308 (T308) and by mTORC2 on serine 473 (S473) [153]. AKT can then act on a variety of downstream effectors including the FOXO1 (Forkhead Box 1) transcription factor and TSC (tuberous sclerosis complex), which releases the inhibitory effect of the complex on mTORC1 [154].

TIE2 directly activates PI3K through recruitment of p85 that is at least partially achieved by docking on the carboxyl tail [155]. Further activation of the pathway to AKT has been shown in many studies [156–158] and measurement of the levels of phosphorylated AKT is considered an important readout of TIE2 activity in EC. Furthermore, activated TIE2 has been shown to increase phosphorylation of mTORC1 target proteins, including S6 kinase [159], as well as FOXO1, whose phosphorylation inactivates it and prevents its regulation of genes important for angiogenesis and vascular remodeling [156]. Together, activation of the PI3K/AKT/mTOR signaling components implicate the TIE2 axis in regulation of EC growth, survival, proliferation, and migration, all cellular phenotypes that are important both for physiological and pathological vessel development. Importantly, this pathway can be effectively inhibited by rapamycin, which is known to inhibit mTORC1 but also can inhibit mTORC2 in some cell types upon chronic treatment [90]. Due to the ability of mTORC2 to phosphorylate and activate AKT, rapamycin treatment in the context of activated TIE2 will reduce AKT phosphorylation at S473, the site acted on by mTORC2 [92].

### MAPK/ERK signaling

The MAPK/ERK signaling cascade is also a highly conserved pathway that is typically involved in cellular proliferation. As with the PI3K pathway, it often begins with mitogenic extracellular signals that are received by transmembrane RTK, including TIE2. Activation of the receptor then recruits adaptor molecules, such as Grb2 (growth factor receptor-binding protein 2) and SOS (son of sevenless), which acts as a guanine exchange factor to promote activation of a Ras protein [160]. Ras can then activate downstream effectors, such as Raf, which initiates a phosphorylation cascade through various MAPK (mitogen activated kinase) components. While there are at least four MAPK cascades with various effector proteins, the one most studied in the context of TIE2 signaling is the MEK/ERK cascade. ERK1/2 (extracellular regulated kinases 1/2) are the terminal effectors of this cascade and phosphorylation/activation of ERK stimulates nuclear translocation and phosphorylation of downstream targets [160].

Phosphorylation of ERK is known to be induced downstream of Angpt1-stimulated activation of TIE2 [159, 161–163] and in cells that express hyperactive mutant TIE2 [78, 91, 95]. While the exact mechanism of MAPK pathway activation downstream of TIE2 has not been exactly defined, protein binding experiments indicate that Grb2 likely interacts with phosphotyrosines present on activated TIE2 [155]. However, unlike PI3K signaling, the downstream effectors and phenotypic consequences of TIE2/MAPK signaling are much less well defined. One study suggests that MAPK inhibition in VM-mutant TIE2 cells can rescue abnormal extracellular matrix deposition (ECM) and aberrant activation of the plasmin proteolytic system [91], although these results have not yet translated into pre-clinical or clinical studies of MAPK inhibitors to treat VM. While not yet tested in VM patients, trametinib is a MEK inhibitor that has been used in some vascular anomaly patients with known mutations in the MAPK/ERK pathway [164, 165] and potentially could be used to target this pathway in VM.

### ABL signaling

Finally, c-ABL (Abelson kinase) has been shown to be hyperactivated downstream of mutant TIE2 and inhibition of c-ABL with ponatinib (or genetic knockdown of c-ABL and its paralog ARG) reduced levels of phospho-ERK and phospho-AKT in TIE2-mutant EC [95]. However, inhibition of ABL reduced TIE2 expression and signaling, raising the possibility that a feedback loop exists between c-ABL and TIE2 and making it difficult to ascertain if/how c-ABL could be signaling to downstream PI3K/AKT and MAPK/ERK signaling effectors [95]. This is supported by previous studies in Abl/Arg knockout mice which demonstrated bidirectional signaling between TIE2 and ABL, in which ABL kinases are activated downstream of TIE2 but are also required to maintain TIE2 expression [166]. While it is unclear how exactly c-ABL could contribute to VM pathogenesis, it has been shown that c-ABL is important for EC motility and adhesion, likely due to its ability to affect cytoskeletal signaling effectors [167]. In zebrafish, a possible link between ABL signaling and vessel lumen size has also been established [168]. Therefore, while additional investigation is needed, it is possible that c-ABL is both an effector and regulator of TIE2 signaling and it may be able to modulate vessel size through its role in regulating the cytoskeleton, possibly implicating c-ABL as an important effector in mutant TIE2-driven VM pathogenesis. Recently, a study has shown that nanoparticles containing both rapamycin and ponatinib were effective in a xenograft model of TIE2-driven VM [169], suggesting that c-ABL inhibitors may be a potential therapeutic option for VM patients.

## TIE2 in vascular physiology

Many lines of evidence suggest that TIE2 signaling is critical for normal vascular growth and function, including embryonic vascular development, angiogenesis, and adult vascular quiescence. On the flipside, the indispensable role of TIE2 in EC biology also facilitates pathological effects when TIE2 is mutated, such as with the TIE2 hyperactivating mutations that occur in VM. Understanding how TIE2 signaling functions in vascular physiology is essential to understanding how those functions change or are co-opted in pathological processes.

### Vascular development

The role of TIE2 signaling in vascular development became apparent from early studies performing genetic knockout of TIE2 signaling components in mice. Dumont and colleagues (1994) were the first to show that homozygous null *Tek* mutations or expression of a Tie2 dominant-negative mutation caused embryonic lethality by embryonic day (E) 9.5–10.5 [170]. At the cellular level, they found that the early steps of vascular development did occur (with the appearance of EC), but the number of EC decreased prior to embryonic death, implicating that Tie2 is not necessary for establishment of the EC lineage, but it is required for EC survival. This study was followed by additional experiments published in 1995 that sought to study Tie1. Interestingly, Tie1 homozygous null mice died after E13.5, with some surviving until birth but dying soon after [171]. As opposed to Tie2 knockout, the Tie1 mutants did have a vascular network, but the endothelial barrier was compromised, leading to edema and hemorrhage. Furthermore, this same study re-examined the effects of Tie2 knockout and formed additional conclusions that loss of Tie2 impaired normal EC sprouting and remodeling [171]. Further work on Tie1-deleted mice clarified that the lymphatic system is especially sensitive, with mutant mice developing embryonic defects in lymphatic patterning and drainage [172].

More recent work has further clarified the specific role of TIE2 in venous development. Chu and colleagues found that Tie2 is more highly expressed in veins during embryogenesis and *Tek* null mouse embryos had disrupted development of venous structures [173]. At the molecular level, they also found that Tie2 likely determines and maintains venous identity through regulation of COUP-TFII, a master transcription factor for venous differentiation. However, tissue context was important in these studies, as loss of Tek abrogated vein development in the skin but merely resulted in abnormal vein/artery alignment in mesenteries. Further contributing to our understanding of this pathway, a study by Li-Villarreal and colleagues found that the FOXO1 transcription factor promotes arterial specification and vascular remodeling in the mouse embryonic yolk sac [174]. Previous literature has established that TIE2 activation of AKT results in phosphorylation and nuclear exclusion of FOXO1 [93]. Additionally, TIE1 could synergize with TIE2 to drive venous specification [175], while deficiency of TIE1 disrupted embryonic vein formation and decreased expression of TIE2 and COUP-TFII. Furthermore, loss of COUP-TFII decreased expression of TIE1 and TIE2, implying that a positive feedback loop exists between these factors to promote venous differentiation. Together, these studies suggest a model in which TIE2 activity promotes venous identify via COUP-TFII and inhibits arterial identify through inhibition of FOXO1, which is supported by expression of TIE1.

The role of Angpt1 in potentiating Tie2 signaling in development was also investigated in mouse knockout models. Full knockout of Angpt1 resulted in embryonic lethality around E12.5 with vascular defects that mimicked those seen in the Tie2 knockout models [176]. Importantly, very similar defects in cardiac structure (abnormal endocardium and trabeculation) were observed, implicating Tie2 in regulation of endocardial development. However, the difference in time of demise between the two models is not entirely clear, but it is possible that activation by other Angpt ligands (such as Angpt2) or transfer of maternal ligands could play a role. More recent studies have expanded on the effects of Angpt1 knockout, which surprisingly showed using a conditional knockout system that deletion of Angpt1 after E13.5 did not cause any overt phenotypes [177]. However, upon injury or stress, Angpt1 loss increased tissue damage through excessive fibrosis and angiogenesis, implicating that although continuous Angpt1 signaling is not necessary for quiescent vasculature, it is necessary to act as a break on angiogenesis upon injury.

Angpt2 knockout revealed very different effects during embryonic development, which is perhaps expected due to its context-dependent roles as an agonist and antagonist of TIE2. Mice that are homozygous for deletion of Angpt2 survived until birth, but died before two weeks of age due to lymphatic dysfunction [178], indicating that it is dispensable for development of the blood vasculature. However, they also found that Angpt2-null mice displayed defects in angiogenic remodeling of the eye vasculature, indicating its role in postnatal vascular remodeling upon stimulus. Furthermore, Angpt2 loss impaired lymphatic development, which could be rescued by Angpt1, indicating that Angpt2 may function more as an agonist within this tissue. Moreover, overexpression of Angpt2 during early development mimicked many of the blood vascular phenotypes of Angpt1 or TIE2 loss, revealing its primary role as an antagonist at this stage [179].

### Vascular quiescence and angiogenesis

The role of TIE2 in maintaining vascular quiescence (in which EC maintain stable, nonleaky blood vessels that neither grow nor regress) has been appreciated since it was discovered that tyrosine-phosphorylated TIE2 was present in adult quiescent blood vessels [180]. TIE2 signaling through the PI3K/AKT pathway is thought to be an important contributor to vessel stabilization, as this signaling pathway has been shown to counteract apoptosis and promote survival in EC [181]. Furthermore, Angpt1 stimulation of PI3K signaling reduces expression and release of Angpt2, which can be associated with TIE2 signaling inhibition and vessel destabilization [182], and Angpt1/TIE2 signaling is also known to be anti-inflammatory in EC [183].

The first evidence that TIE2 was involved in angiogenesis (the formation of new blood vessels from existing ones) is that TIE2 expression and activation is increased over baseline in areas of active vessel growth, such as in the ovary and in wound healing [180]. However, the effect of TIE2 signaling in angiogenic tissues is also modulated by the presence of VEGF signaling, the most potent inducer of angiogenesis. Blockade of VEGF in preclinical mouse experiments showed that vascular normalization (the formation of stable, nonleaky vessels that are anti-tumorigenic) is enhanced by upregulation of Angpt1 [184]. Therefore, TIE2 signaling may be angiogenic only in settings of stimulation of additional signaling pathways. Additional contextual signals are provided by the cell type(s) that express TIE2. Interestingly, tip cells (the leading cells in formation of new blood vessels) actually appear to downregulate TIE2, while the stalk cells (which follow behind the tip cell) express TIE2 [185]. Therefore, while TIE2 signaling is necessary for the overall process of tip and stalk cell communication and migration, it may be downregulated on the angiogenic leading cell to overcome the overall quiescent effect of the signaling pathway.

## Mechanisms of venous malformation pathogenesis

While the full cellular and molecular mechanisms that drive VM formation downstream of mutant TIE2 have not been fully elucidated, investigation of experimental models and clinical specimens are beginning to reveal potential answers. In addition, discoveries made in understanding the pathogenic mechanisms of other types of vascular anomalies can be used as examples to speculate on how similar mechanisms may be taking place in VM. Several topics of interest in the pathogenesis of VM are discussed in greater detail in the following subsections.

### Proliferation

While historically, when compared to vascular tumors, EC proliferation in VM has not been considered pathological, recent evidence suggests that VM lesions do contain proliferative EC, which can contribute to blood vessel enlargement. Immunohistochemical analysis of VM patient samples revealed the presence of lesional EC that are positive for Ki67, a marker of cell proliferation [186]. Furthermore, in vitro models of TIE2 p.L914F-mutant EC [95] and PIK3CA-mutant EC [114] have shown that these cells demonstrate increased proliferation rates. Finally, increased EC proliferation has also been associated with VM lesion formation in *PIK3CA*-mutant murine VM models [51, 52]. While increased proliferation is seen in systems with PI3K mutation, showing that activation of this pathway is sufficient to drive this cellular phenotype, the role of TIE2 downstream effectors in this phenomenon are less clearly known. TIE2 p.L914F is known to drive both PI3K/AKT and MAPK/ERK signaling (as described previously), and both have been implicated in EC proliferation and survival. However, the relative contribution of these pathways to the increased proliferation in TIE2-mutant VM EC is not known. Treatment with rapamycin [51] or alpelsib [52] in *PIK3CA*-mutant VM models has been shown to reduce cellular proliferation in VM lesions, indicating that the PI3K/AKT/mTOR pathway is almost certainly playing a role. However, it is unknown whether MAPK/ERK inhibition will reduce EC proliferation in TIE2 mutant conditions. Additional work in vitro and in vivo will be needed to determine how proliferation plays a role in VM lesion formation and progression and whether targeting of each pathway (or both) will successfully counteract this phenotype.

### Migration and lumen formation

Furthermore, EC migration is an important component of blood vessel formation and remodeling [187]. In normal venous physiology, proliferation and directed migration are coupled. During in vivo conditions of vascular growth (such as during development or neovascularization), venous EC are more responsive than arterial EC to stimulatory signals. They undergo increased proliferation and then migrate directionally against blood flow [188]. While these results must be interpreted in context, as these phenomena should be investigated in different tissues and stages of growth, they provide a framework in which we should consider directed migration of venous EC as an essential component of normal vessel function. In terms of VM pathogenesis, in vitro monolayer and 3D experiments using transduced HUVEC have demonstrated that mutant TIE2 EC have increased migration rates but lack normal polarity, resulting in more random movement [94]. In 3D models of EC and adipose stem cell co-cultures, TIE2 p.L914F mutant EC again displayed decreased polarization during tube formation and lacked filipodia and sprouting activity [189]. Increased migration rates of TIE2-mutant EC have also been confirmed in iPSC and CRISPR generated cell models, where transcriptomic analyses have illustrated global upregulation of genes related to cell migration [104]. Therefore, the increased but random movement seen in in vitro models of TIE2-mutant EC may interfere with the normal process of directed venous EC migration and contribute to abnormal vessel morphogenesis. However, experiments examining EC migration in the context of in vivo VM lesions will be necessary to confirm this hypothesis.

While the findings above confirm the phenotypic alterations to EC migration and polarity upon TIE2 mutation, much less is known about the molecular changes that could be driving these phenotypes. However, there is recent literature that examines how cytoskeletal signaling effectors can affect EC migration and lumen formation and how these pathways are changed in *PIK3CA* mutant cells. It was previously established by the Davis lab that the Rho GTPases CDC42 and RAC1 are important for EC lumen formation in 3D matrices [190]. Furthermore, regulation of actin-myosin contractility via RhoA modulation has been shown to control lumen size via regulation of EC spreading [191]. Linking these concepts together, Laviña and colleagues showed that in a murine model, retinal EC that lost expression of CDC42 showed polarity and migration defects that contributed to vascular malformations with increased vessel size [192]. Similarly, Castro and colleagues demonstrated that another murine model of EC-specific CDC42 deletion resulted in a CCM-like vascular phenotype associated with loss of EC dispersion (migration), sprouting, and filipodia formation [193]. In *PIK3CA*-mutant EC, RAC1 was found to be elevated and was thought to contribute to abnormal migration and vessel expansion [103]. In total, these studies illustrate the essential role of Rho GTPase signaling in vessel morphogenesis, particularly cell polarity, migration, and lumen formation. Therefore, these are pathways that are likely to be impacted in TIE2-driven VM formation and further research is warranted.

### Hemodynamics and flow sensing

EC migration is guided by frictional forces imposed by blood flow (often termed shear stress). The direction of blood flow is a major influence in determining the direction of EC migration [187] and shear stress signaling is also thought to contribute to suppression of EC proliferation [194]. In general, EC sensing of the shear stress set point (the amount of stress that is perceived as normal for the vessel type [195]) is thought to help maintain vessel quiescence with normal blood flow but also to promote vessel remodeling when flow is abnormal [196]. This is especially relevant to the pathogenesis of vascular malformations, as these abnormal vessels are known to experience highly disturbed blood flow. In VMs, the flow is very slow compared to normal veins and is also turbulent, due to the abnormal vessel morphology.

There is recent but limited evidence that VM-causative mutations impair the ability of EC to appropriately respond to stimulated in vitro shear stress. Using iPSC-derived TIE2-mutant EC in a microfluidic model, Lazovic and colleagues demonstrated that these cells experience reduced alignment to flow conditions and, interestingly, increased cell area, particularly in conditions of low flow [104]. In a model of *PIK3CA* mutation, flow induced alignment and elongation of wild-type cells, while mutant EC failed to undergo these processes [197], which could impair migration of mutant EC. These results are especially interesting considering that wild-type TIE2 is activated by flow [198, 199]. Thus, an important question that remains is whether VM mutations, at baseline, mimic the signaling that would normally occur upon flow sensing but without directionality, therefore making these EC unable to respond when flow is induced. Another possibility is that the mutations interfere with other flow-sensing proteins and pathways, which would also explain their unresponsiveness to this signal. More work in models that incorporate flow and shear stress is necessary to investigate these possibilities.

To summarize the possible pathogenic mechanisms that were discussed in this section and the previous two sections, a combination of increased EC proliferation but impaired migration (especially in the context of impaired flow sensing) would cause the accumulation of EC within mutant vessels, providing a possible explanation for vessel enlargement.

### Extracellular environment

The ability of mutant EC to interact with surrounding cells and matrix components could contribute to VM pathology. It is well established from VM patient histology that mural cells are abnormally recruited to lesional blood vessels [41, 42]. This has also been shown in 3D in vitro models, where TIE2 p.L914F HUVEC failed to recruit pericytes in the lumen formation assay [94]. One mechanism proposed for this in the literature is that TIE2 p.L914F mutant EC produce less PDGFB (platelet-derived growth factor subunit B), a chemical factor that serves to recruit vSMC and other mural cells to EC-lined vessels [43]. However, there has yet to be conclusive evidence that, first, PDGFB is the only factor responsible for failed mural recruitment to VM vessels and, second, that restoring mural cell recruitment would rescue vessel enlargement. However, recent progress has been made using newly developed in vitro systems to interrogate the changes that occur in perivascular cells when in contact with TIE2-mutant EC. Ansarizadeh and colleagues generated a co-culture system (mentioned briefly above in Sect. 7.2) with EC and adipose-derived stem cells, which are angiogenic vascular support cells [189]. Like previous results, they show that TIE2 mutant EC experience reduced coverage of αSMA (alpha smooth muscle actin) positive perivascular cells. However, they take their analysis one step further by performing transcriptomic analysis of perivascular αSMA positive cells, revealing that exposure to TIE2 mutant EC causes changes to regulation of perivascular cell migration, cytoskeleton, and ECM [189]. While these results do not conclusively determine the changes that are happening in VM perivascular cells, they provide further directions for investigation of these cells across experimental models of VM.

Beyond cellular components of the extracellular environment, ECM and basement membrane components help to support a normal vessel wall and electron microscopy analysis of VM patient tissue samples have suggested that the ECM in lesional vessels is disorganized and sparse [91, 200]. A recent study has analyzed this phenomenon in more detail using spatial transcriptomics, where they showed that TIE2 mutant patient VM lesions had a greater upregulation of genes related to extracellular matrix organization than normal and *PIK3CA* mutant VM vessels [65]. Collagen too has been found to be reduced in VM patient samples [201]. Analysis of TIE2 mutant cell lines suggests that these changes observed in patient samples could be due to decreased deposition of ECM components, such as fibronectin, or increased production of proteolytic enzymes [91]. Microarray analysis of a TIE2 mutant cell line had also shown a strong upregulated signature of metalloendopeptidase activity, which is known to be involved in ECM cleavage [43]. More recently, a study of TIE2 mutant patient samples and engineered cell lines showed that these cells have increased production of extracellular vesicles (EVs) that additionally carry matrix metalloproteinase 14 (MMP14) [202], contributing to our understanding of how VM mutant EC may interact with their environment. However, experiments in vivo have not yet been performed that show that rescue of the ECM defects will correct vessel enlargement.

### Immune components

While immune alterations in VM seem logical considering that hemodynamic stasis and thrombosis are well-established phenotypes, the role of immune cell infiltration and coagulation signaling has not been studied in TIE2 mutant VM. However, findings from *PIK3CA* mutant VM mouse models and from other models of vascular anomalies inform how these pathways may go awry in TIE2-driven VM. Petkova and colleagues utilized a *PIK3CA*-driven murine model of VM to show that cutaneous VM lesions may experience increased infiltration of neutrophils and B cells [116]. However, the authors also found a much stronger immune signature in the context of *PIK3CA*-driven lymphatic overgrowth and discovered that pro-inflammatory signaling contributed to macrophage recruitment, which supported pathological lymphangiogenesis. The pathological role of the weaker immune signature in blood VM lesions was not explored. Adding to this work, Kraft and colleagues later investigated the *PIK3CA*-driven VM model and discovered that lesional EC had transcriptional signatures reflective of post-capillary venous EC, which are known to be involved in leukocyte extravasation [117].

Beyond VM, macrophage recruitment and accumulation has also been observed in murine models of AVM [203]. In CCM, as published by Tang and colleagues, microbiome-derived bacterial components were responsible for activating immune signaling receptors on EC within CCM lesions, which accelerated disease growth [204]. More recent work has also demonstrated the role of stromal cells in CCM immune phenotypes by reporting that brain astrocytes in regions of CCM lesions become inflammatory and contribute to immune cell recruitment (termed neuroinflammation) [205]. The resulting inflammatory milieu, involving astrocytes, EC, and immune cells, promotes hypoxia signaling and angiogenesis, contributing to lesion progression and immunothrombosis. In total, these results provide hints that immune trafficking, extravasation, and signaling may be involved in VM pathogenesis, as they are involved in pathogenesis of other vascular lesions, but additional work is needed to determine which immune populations could be playing a role and how VM-causative mutations in *PIK3CA* and TIE2 contribute or respond to alterations in immune signaling.

### TIE2 signaling in non-TIE2 mutant vascular anomalies

Beyond the role of mutant TIE2 in VM, recent evidence indicates that canonical TIE2 signaling is also implicated in other types of vascular anomalies. First, in a *PIK3CA*-mutant model of VM, Kraft and colleagues found increased levels of TIE2 phosphorylation within VM lesional vessels and treatment with a TIE2 inhibitor was effective in reducing lesion growth [117]. They hypothesized that a positive feedback loop formed, wherein PI3K/AKT signaling reduced expression of ANGPT2 (which can act as a TIE2 antagonist), increasing TIE2 activation by ANGPT1, therefore feeding increased PI3K/AKT signaling. Conversely, ANGPT2 is thought to play a role in TIE2 activation in the context of GNAQ p.R183Q mutation. Here, Liu and colleagues found that ANGPT2 production by GNAQ mutant cells contributed to TIE2 activation and cell survival [206], illustrating the complex and context-dependent nature of ANGPT activation of TIE2.

TIE2 signaling has also been implicated in CCM, however by a different mechanism. Li and colleagues found that TIE2 signaling was increased in mouse brain CCM lesions, genetic or pharmacological inhibition of TIE2 reduced CCM formation, and that these effects were likely to be mediated by increased TIE2 expression via KLF2/4 activity downstream of CCM protein loss-of-function [207]. A similar mechanism may also play a role in AVM, as SMAD4 loss changes the fluid shear stress set point so that increased shear stress sensing causes upregulation of KLF4 and therefore TIE2 [208]. Together, these results emphasize the importance of understanding the mechanisms and effectors of TIE2 signaling in many types of vascular anomalies, not just venous malformations.

## Conclusions

Each disorder within the vascular anomaly family is defined by its own clinical characteristics, causative genetic mutations, and pathogenic mechanisms. Understanding these factors is essential for diagnosis and treatment of patients, especially as targeted, non-invasive therapies are becoming increasingly accessible. For VM specifically, discovery of the causative mutations in the *TEK* (TIE2) and *PIK3CA* (PI3K p110 subunit) genes have allowed for the development of in vitro and in vivo model systems that recapitulate the signaling and cellular processes that drive VM pathogenesis. Investigation of these pathways has yielded progress in the identification of new targeted therapies for patients, although there is still significant need for improved options that are more efficacious without compromising quality of life. Greater understanding of TIE2 signaling, by means of improved model systems addressing the role of TIE2 both during normal physiology and in the context of VM pathology, will better allow clinicians and researchers to find these options.

## References

[1] Wassef M, Blei F, Adams D et al (2015) Vascular anomalies classification: recommendations from the international society for the study of vascular anomalies. Pediatrics 136(1):e203–e214. 10.1542/peds.2014-3673 26055853 10.1542/peds.2014-3673

[2] Goldenberg DC, Vikkula M, Penington A et al (2025) Updated classification of vascular anomalies. A living document from the international society for the study of vascular anomalies classification group. J Vasc Anomalies 6(2):e113. 10.1097/JOVA.0000000000000113 10.1097/JOVA.0000000000000113 PMC12237419 40636925

[3] Nosher JL, Murillo PG, Liszewski M, Gendel V, Gribbin CE (2014) Vascular anomalies: a pictorial review of nomenclature, diagnosis and treatment. World J Radiol 6(9):677–692. 10.4329/wjr.v6.i9.677 25276311 10.4329/wjr.v6.i9.677 PMC4176785

[4] Enjolras O (1997) Classification and management of the various superficial vascular anomalies: hemangiomas and vascular malformations. J Dermatol 24(11):701–710. 10.1111/j.1346-8138.1997.tb02522.x 9433027 10.1111/j.1346-8138.1997.tb02522.x

[5] Enjolras O, Mulliken JB (1997) Vascular tumors and vascular malformations (new issues). Adv Dermatol 13:375–423 9551150

[6] Munden A, Butschek R, Tom WL et al (2014) Prospective study of infantile haemangiomas: incidence, clinical characteristics and association with placental anomalies. Br J Dermatol 170(4):907–913. 10.1111/bjd.12804 24641194 10.1111/bjd.12804 PMC4410180

[7] Drolet BA, Trenor CC, Brandão LR et al (2013) Consensus-derived practice standards plan for complicated kaposiform hemangioendothelioma. J Pediatr 163(1):285–291. 10.1016/j.jpeds.2013.03.080 23796341 10.1016/j.jpeds.2013.03.080

[8] Schrenk S, Bischoff LJ, Goines J et al (2023) MEK inhibition reduced vascular tumor growth and coagulopathy in a mouse model with hyperactive GNAQ. Nat Commun. 10.1038/s41467-023-37516-7 37024491 10.1038/s41467-023-37516-7 PMC10079932

[9] Lim YH, Bacchiocchi A, Qiu J et al (2016) GNA14 somatic mutation causes congenital and sporadic vascular tumors by MAPK activation. Am J Hum Genet 99(2):443–450. 10.1016/j.ajhg.2016.06.010 27476652 10.1016/j.ajhg.2016.06.010 PMC4974082

[10] Ayturk UM, Couto JA, Hann S et al (2016) Somatic activating mutations in GNAQ and GNA11 are associated with congenital hemangioma. Am J Hum Genet 98(4):789–795. 10.1016/j.ajhg.2016.03.009 27058448 10.1016/j.ajhg.2016.03.009 PMC4833432

[11] Meis-Kindblom JM, Kindblom LG (1998) Angiosarcoma of soft tissue: a study of 80 Cases. Am J Surg Pathol 22(6):683 9630175 10.1097/00000478-199806000-00005

[12] Torrence D, Antonescu CR (2022) The genetics of vascular tumors: an update. Histopathology 80(1):19–32. 10.1111/his.14458 34958509 10.1111/his.14458 PMC8950088

[13] Derdeyn CP, Zipfel GJ, Albuquerque FC et al (2017) Management of brain arteriovenous malformations: a scientific statement for healthcare professionals from the American Heart Association/American Stroke Association. Stroke 48(8):e200–e224. 10.1161/STR.0000000000000134 28642352 10.1161/STR.0000000000000134

[14] Al-Olabi L, Polubothu S, Dowsett K et al (2018) Mosaic RAS/MAPK variants cause sporadic vascular malformations which respond to targeted therapy. J Clin Invest 128(4):1496. 10.1172/JCI98589 29461977 10.1172/JCI98589 PMC5873857

[15] Lapinski PE, Doosti A, Salato V, North P, Burrows PE, King PD (2018) Somatic second hit mutation of RASA1 in vascular endothelial cells in capillary malformation-arteriovenous malformation. Eur J Med Genet 61(1):11–16. 10.1016/j.ejmg.2017.10.004 29024832 10.1016/j.ejmg.2017.10.004 PMC5766414

[16] Amyere M, Revencu N, Helaers R et al (2017) Germline loss-of-function mutations in EPHB4 cause a second form of capillary malformation-arteriovenous malformation (CM-AVM2) deregulating RAS-MAPK signaling. Circulation 136(11):1037–1048. 10.1161/CIRCULATIONAHA.116.026886 28687708 10.1161/CIRCULATIONAHA.116.026886

[17] Sun Y, Su L, Zhang Y et al (2023) Genotype–phenotype and genotype–outcome analysis of capillary malformation–arteriovenous malformation. J Vasc Anomal 4(3):e052. 10.1097/JOVA.0000000000000052

[18] McDonald J, Bayrak-Toydemir P, DeMille D, Wooderchak-Donahue W, Whitehead K (2020) Curaçao diagnostic criteria for hereditary hemorrhagic telangiectasia is highly predictive of a pathogenic variant in *ENG* or *ACVRL1* (HHT1 and HHT2). Genet Med 22(7):1201–1205. 10.1038/s41436-020-0775-8 32300199 10.1038/s41436-020-0775-8

[19] Plauchu H, De Chadarévian JP, Bideau A, Robert JM (1989) Age-related clinical profile of hereditary hemorrhagic telangiectasia in an epidemiologically recruited population. Am J Med Genet 32(3):291–297. 10.1002/ajmg.1320320302 2729347 10.1002/ajmg.1320320302

[20] Gupta A, Kozakewich H (2011) Histopathology of vascular anomalies. Clin Plast Surg 38(1):31–44. 10.1016/j.cps.2010.08.007 21095470 10.1016/j.cps.2010.08.007

[21] Shirley MD, Tang H, Gallione CJ et al (2013) Sturge–Weber syndrome and port-wine stains caused by somatic mutation in GNAQ. N Engl J Med 368(21):1971–1979. 10.1056/NEJMoa1213507 23656586 10.1056/NEJMoa1213507 PMC3749068

[22] Mäkinen T, Boon LM, Vikkula M, Alitalo K (2021) Lymphatic malformations: genetics, mechanisms and therapeutic strategies. Circ Res 129(1):136–154. 10.1161/CIRCRESAHA.121.318142 34166072 10.1161/CIRCRESAHA.121.318142

[23] Luks VL, Kamitaki N, Vivero MP et al (2015) Lymphatic and other vascular malformative/overgrowth disorders are caused by somatic mutations in PIK3CA. J Pediatr 166(4):1048-1054.e5. 10.1016/j.jpeds.2014.12.069 25681199 10.1016/j.jpeds.2014.12.069 PMC4498659

[24] Ozeki M, Aoki Y, Nozawa A et al (2019) Detection of NRAS mutation in cell-free DNA biological fluids from patients with kaposiform lymphangiomatosis. Orphanet J Rare Dis 14(1):215. 10.1186/s13023-019-1191-5 31511039 10.1186/s13023-019-1191-5 PMC6737666

[25] Nozawa A, Ozeki M, Niihori T, Suzui N, Miyazaki T, Aoki Y (2020) A somatic activating KRAS variant identified in an affected lesion of a patient with Gorham-Stout disease. J Hum Genet 65(11):995–1001. 10.1038/s10038-020-0794-y 32591603 10.1038/s10038-020-0794-y

[26] Zhou W, Zeng L, Chen Y, Zhang F (2026) Pulmonary endothelial heterogeneity shapes divergent molecular vulnerabilities under SMAD4 deficiency. Vessel Plus. 10.20517/2574-1209.2025.143

[27] Banerjee K, Lin Y, Gahn J et al (2023) SMAD4 maintains the fluid shear stress set point to protect against arterial-venous malformations. J Clin Invest 133(18):e168352. 10.1172/JCI168352 37490341 10.1172/JCI168352 PMC10503796

[28] Lin Y, Hashemi Z, Zhang Q et al (2026) Flow-induced Klf4-Akt signaling links EC cycling to mural cell defects in arterial-venous malformations. Theranostics 16(9):4905–4922. 10.7150/thno.121154 41799188 10.7150/thno.121154 PMC12964382

[29] Ola R, Künzel SH, Zhang F et al (2018) SMAD4 prevents flow induced arteriovenous malformations by inhibiting casein kinase 2. Circ 138(21):2379–2394. 10.1161/CIRCULATIONAHA.118.033842 10.1161/CIRCULATIONAHA.118.033842 PMC6309254 29976569

[30] Galeffi F, Snellings DA, Wetzel-Strong SE et al (2022) A novel somatic mutation in GNAQ in a capillary malformation provides insight into molecular pathogenesis. Angiogenesis 25(4):493–502. 10.1007/s10456-022-09841-w 35635655 10.1007/s10456-022-09841-w PMC9529792

[31] Vikkula M, Boon LM, Mulliken JB (2001) Molecular genetics of vascular malformations. Matrix Biol 20(5–6):327–335. 10.1016/S0945-053X(01)00150-0 11566267 10.1016/s0945-053x(01)00150-0

[32] Penington A, Phillips RJ, Sleebs N, Halliday J (2023) Estimate of the prevalence of vascular malformations. J Vasc Anom 4(3):e068. 10.1097/JOVA.0000000000000068

[33] Gallagher JR, Carroll S, Martini J, Kelly M, Buethe MG (2025) Currently managed US prevalence of cutaneous venous malformations (cVMs): a nationally representative, retrospective, real-world, subject-blinded, physician-observational probability study. Orphanet J Rare Dis 20:504. 10.1186/s13023-025-03995-8 41057921 10.1186/s13023-025-03995-8 PMC12502304

[34] Hassanein AH, Mulliken JB, Fishman SJ, Alomari AI, Zurakowski D, Greene AK (2012) Venous malformation: risk of progression during childhood and adolescence. Ann Plast Surg 68(2):198–201. 10.1097/SAP.0b013e31821453c8 21629093 10.1097/SAP.0b013e31821453c8

[35] Behravesh S, Yakes W, Gupta N et al (2016) Venous malformations: clinical diagnosis and treatment. Cardiovasc Diagn Ther 6(6):557–569. 10.21037/cdt.2016.11.10 28123976 10.21037/cdt.2016.11.10 PMC5220204

[36] Vogel SA, Hess CP, Dowd CF et al (2013) Early versus later presentations of venous malformations: where and why? Pediatr Dermatol 30(5):534–540. 10.1111/pde.12162 23679583 10.1111/pde.12162

[37] Dompmartin A, Ballieux F, Thibon P et al (2009) Elevated D-dimer level is diagnostic for venous malformations. Arch Dermatol 145(11):1239–1244. 10.1001/archdermatol.2009.296 19917952 10.1001/archdermatol.2009.296 PMC5561655

[38] Hu L, Chen H, Yang X et al (2019) Risk factors associated with pain in patients with venous malformations of the extremities. Vasc Med 24(1):56–62. 10.1177/1358863X18802007 30340449 10.1177/1358863X18802007

[39] Dompmartin A, Acher A, Thibon P et al (2008) Association of localized intravascular coagulopathy with venous malformations. Arch Dermatol 144(7):873–877. 10.1001/archderm.144.7.873 18645138 10.1001/archderm.144.7.873 PMC5572565

[40] Mazoyer E, Enjolras O, Bisdorff A, Perdu J, Wassef M, Drouet L (2008) Coagulation disorders in patients with venous malformation of the limbs and trunk: a case series of 118 patients. Arch Dermatol 144(7):861–867. 10.1001/archderm.144.7.861 18645137 10.1001/archderm.144.7.861

[41] Fernandez-Flores A, Cassarino D, Colmenero I (2023) Vascular malformations: a histopathologic and conceptual appraisal. Actas Dermo-Sifiliogr 114(3):213–228. 10.1016/j.ad.2022.10.035 10.1016/j.ad.2022.10.035 36309042

[42] Limaye N, Wouters V, Uebelhoer M et al (2009) Somatic mutations in the angiopoietin-receptor TIE2 can cause both solitary and multiple sporadic venous malformations. Nat Genet 41(1):118–124. 10.1038/ng.272 19079259 10.1038/ng.272 PMC2670982

[43] Uebelhoer M, Nätynki M, Kangas J et al (2013) Venous malformation-causative TIE2 mutations mediate an AKT-dependent decrease in PDGFB. Hum Mol Genet 22(17):3438–3448. 10.1093/hmg/ddt198 23633549 10.1093/hmg/ddt198 PMC3736867

[44] Jinnin M, Medici D, Park L et al (2008) Suppressed NFAT-dependent VEGFR1 expression and constitutive VEGFR2 signaling in infantile hemangioma. Nat Med 14(11):1236–1246. 10.1038/nm.1877 18931684 10.1038/nm.1877 PMC2593632

[45] Zafar A, Quadri SA, Farooqui M et al (2019) Familial cerebral cavernous malformations. Stroke 50(5):1294–1301. 10.1161/STROKEAHA.118.022314 30909834 10.1161/STROKEAHA.118.022314 PMC6924279

[46] Brouillard P, Boon LM, Mulliken JB et al (2002) Mutations in a novel factor, glomulin, are responsible for glomuvenous malformations (“glomangiomas”). Am J Hum Genet 70(4):866–874. 10.1086/339492 11845407 10.1086/339492 PMC379115

[47] Boon LM, Mulliken JB, Vikkula M et al (1994) Assignment of a locus for dominantly inherited venous malformations to chromosome 9p. Hum Mol Genet 3(9):1583–1587. 10.1093/hmg/3.9.1583 7833915 10.1093/hmg/3.9.1583

[48] Vikkula M, Boon LM, Carraway KL et al (1996) Vascular dysmorphogenesis caused by an activating mutation in the receptor tyrosine kinase TIE2. Cell 87:1181–1190. 10.1016/s0092-8674(00)81814-0 8980225 10.1016/s0092-8674(00)81814-0

[49] Calvert JT, Riney TJ, Kontos CD et al (1999) Allelic and locus heterogeneity in inherited venous malformations. Hum Mol Genet 8(7):1279–1289. 10.1093/hmg/8.7.1279 10369874 10.1093/hmg/8.7.1279

[50] Wouters V, Limaye N, Uebelhoer M et al (2010) Hereditary cutaneomucosal venous malformations are caused by TIE2 mutations with widely variable hyper-phosphorylating effects. Eur J Hum Genet 18(4):414–420. 10.1038/ejhg.2009.193 19888299 10.1038/ejhg.2009.193 PMC2841708

[51] Castillo SD, Tzouanacou E, Zaw-Thin M et al (2016) Somatic activating mutations in Pik3ca cause sporadic venous malformations in mice and humans. Sci Transl Med 8(332):332ra43. 10.1126/scitranslmed.aad9982 27030595 10.1126/scitranslmed.aad9982 PMC5973268

[52] Castel P, Carmona FJ, Grego-Bessa J et al (2016) Somatic PIK3CA mutations as a driver of sporadic venous malformations. Sci Transl Med 8(332):332ra42. 10.1126/SCITRANSLMED.AAF1164 27030594 10.1126/scitranslmed.aaf1164 PMC4962922

[53] Limaye N, Kangas J, Mendola A et al (2015) Somatic activating PIK3CA mutations cause venous malformation. Am J Hum Genet 97(6):914–921. 10.1016/j.ajhg.2015.11.011 26637981 10.1016/j.ajhg.2015.11.011 PMC4678782

[54] Sikta N, Gooley S, Green TE et al (2025) Improving genetic diagnostic yield in familial and sporadic cerebral cavernous malformations: detection of copy number and deep intronic variants. Hum Mol Genet 34(15):1286–1293. 10.1093/hmg/ddaf077 40401429 10.1093/hmg/ddaf077 PMC12278727

[55] Bergametti F, Denier C, Labauge P et al (2005) Mutations within the programmed cell death 10 gene cause cerebral cavernous malformations. Am J Hum Genet 76(1):42–51. 10.1086/426952 15543491 10.1086/426952 PMC1196432

[56] Queisser A, Seront E, Boon LM, Vikkula M (2021) Genetic basis and therapies for vascular anomalies. Circ Res 129(1):155–173. 10.1161/CIRCRESAHA.121.318145 34166070 10.1161/CIRCRESAHA.121.318145

[57] Knudson AG (1971) Mutation and cancer: statistical study of retinoblastoma. Proc Natl Acad Sci U S A 68(4):820–823. 10.1073/pnas.68.4.820 5279523 10.1073/pnas.68.4.820 PMC389051

[58] Tsutsumi S, Ogino I, Miyajima M et al (2013) Genomic causes of multiple cerebral cavernous malformations in a Japanese population. J Clin Neurosci 20(5):667–669. 10.1016/j.jocn.2012.05.041 23485406 10.1016/j.jocn.2012.05.041

[59] Ren AA, Snellings DA, Su SY et al (2021) PIK3CA and CCM mutations fuel cavernomas through a cancer-like mechanism. Nature 594(7862):271–276. 10.1038/s41586-021-03562-8 33910229 10.1038/s41586-021-03562-8 PMC8626098

[60] Peyre M, Miyagishima D, Bielle F et al (2021) Somatic PIK3CA mutations in sporadic cerebral cavernous malformations. N Engl J Med 385(11):996–1004. 10.1056/NEJMoa2100440 34496175 10.1056/NEJMoa2100440 PMC8606022

[61] Snellings DA, Girard R, Lightle R et al (2022) Developmental venous anomalies are a genetic primer for cerebral cavernous malformations. Nat Cardiovasc Res 1(3):246–252. 10.1038/s44161-022-00035-7 35355835 10.1038/s44161-022-00035-7 PMC8958845

[62] Wang K, Zhang H, He Y et al (2020) Mural cell-specific deletion of cerebral cavernous malformation 3 in the brain induces cerebral cavernous malformations. Arterioscler Thromb Vasc Biol 40(9):2171–2186. 10.1161/ATVBAHA.120.314586 32640906 10.1161/ATVBAHA.120.314586

[63] Lampugnani MG, Orsenigo F, Rudini N et al (2010) CCM1 regulates vascular-lumen organization by inducing endothelial polarity. J Cell Sci 123(7):1073–1080. 10.1242/jcs.059329 20332120 10.1242/jcs.059329

[64] Soblet J, Limaye N, Uebelhoer M, Boon LM, Vikkula M (2013) Variable somatic TIE2 mutations in half of sporadic venous malformations. Mol Syndromol 4(4):179–183. 10.1159/000348327 23801934 10.1159/000348327 PMC3666452

[65] Hirose K, Hori Y, Ozeki M et al (2024) Comprehensive phenotypic and genomic characterization of venous malformations. Hum Pathol 145:48–55. 10.1016/j.humpath.2024.02.004 38367816 10.1016/j.humpath.2024.02.004

[66] Ghasemi R, Corliss MM, Bowling KM et al (2024) Comprehensive analysis of TEK variants in patients with vascular malformations. Clin Genet. 10.1111/cge.14667 39632338 10.1111/cge.14667

[67] Du Z, Liu JL, You YH et al (2021) Genetic landscape of common venous malformations in the head and neck. J Vasc Surg Venous Lymphat Disord 9(4):1007-1016.e7. 10.1016/j.jvsv.2020.11.016 33248299 10.1016/j.jvsv.2020.11.016

[68] Amyere M, Aerts V, Brouillard P et al (2013) Somatic uniparental isodisomy explains multifocality of glomuvenous malformations. Am J Hum Genet 92(2):188–196. 10.1016/j.ajhg.2012.12.017 23375657 10.1016/j.ajhg.2012.12.017 PMC3567282

[69] Sudduth CL, Konczyk DJ, Smits PJ et al (2021) Bockenheimer disease is associated with a TEK variant. Mol Case Stud 7(6):a006119. 10.1101/mcs.a006119 10.1101/mcs.a006119 PMC8751421 34649969

[70] Nahm WK, Moise S, Eichenfield LF et al (2004) Venous malformations in blue rubber bleb nevus syndrome: variable onset of presentation. J Am Acad Dermatol 50(5):101–106. 10.1016/S0190-9622(03)02468-X 15097941 10.1016/s0190-9622(03)02468-x

[71] Soblet J, Kangas J, Nätynki M et al (2017) Blue rubber bleb nevus (BRBN) syndrome is caused by somatic *TEK* (TIE2) mutations. J Invest Dermatol 137(1):207–216. 10.1016/j.jid.2016.07.034 27519652 10.1016/j.jid.2016.07.034

[72] Fruman DA, Rommel C (2014) PI3K and cancer: lessons, challenges and opportunities. Nat Rev Drug Discov 13(2):140–156. 10.1038/nrd4204 24481312 10.1038/nrd4204 PMC3994981

[73] Osborn AJ, Dickie P, Neilson DE et al (2015) Activating PIK3CA alleles and lymphangiogenic phenotype of lymphatic endothelial cells isolated from lymphatic malformations. Hum Mol Genet 24(4):926–938. 10.1093/hmg/ddu505 25292196 10.1093/hmg/ddu505

[74] Reynolds G, Cardaropoli S, Carli D et al (2023) Epidemiology of the disorders of the Pik3ca-related overgrowth spectrum (Pros). Eur J Hum Genet 31(11):1333–1336. 10.1038/s41431-023-01414-9 37365400 10.1038/s41431-023-01414-9 PMC10620148

[75] Bertino F, Chaudry G (2019) Overgrowth syndromes associated with vascular anomalies. Semin Roentgenol 54(4):349–358. 10.1053/j.ro.2019.06.005 31706368 10.1053/j.ro.2019.06.005

[76] Zhang B, He R, Xu Z et al (2023) Somatic mutation spectrum of a Chinese cohort of pediatrics with vascular malformations. Orphanet J Rare Dis 18:261. 10.1186/s13023-023-02860-w 37658401 10.1186/s13023-023-02860-w PMC10474751

[77] Ten Broek RW, Eijkelenboom A, van der Vleuten CJM et al (2019) Comprehensive molecular and clinicopathological analysis of vascular malformations: a study of 319 cases. Genes Chromosomes Cancer 58(8):541–550. 10.1002/gcc.22739 30677207 10.1002/gcc.22739 PMC6594036

[78] Goines J, Li X, Cai Y et al (2018) A xenograft model for venous malformation. Angiogenesis 21(4):725–735. 10.1007/s10456-018-9624-7 29786783 10.1007/s10456-018-9624-7 PMC6203618

[79] Schrenk S, Sherpa C, Bischoff LJ, Cai Y, Boscolo E (2025) Semaphorin 3A and 3F promote lumen expansion in TIE2-mutated venous malformation. Arterioscler Thromb Vasc Biol. 10.1161/ATVBAHA.125.323387 41127911 10.1161/ATVBAHA.125.323387 PMC12616615

[80] Akers AL, Johnson E, Steinberg GK, Zabramski JM, Marchuk DA (2009) Biallelic somatic and germline mutations in cerebral cavernous malformations (CCMs): evidence for a two-hit mechanism of CCM pathogenesis. Hum Mol Genet 18(5):919–930. 10.1093/hmg/ddn430 19088123 10.1093/hmg/ddn430 PMC2640209

[81] Detter MR, Snellings DA, Marchuk DA (2018) Cerebral cavernous malformations develop through clonal expansion of mutant endothelial cells. Circ Res 123(10):1143–1151. 10.1161/CIRCRESAHA.118.313970 30359189 10.1161/CIRCRESAHA.118.313970 PMC6205520

[82] Johnson CM, Navarro OM (2017) Clinical and sonographic features of pediatric soft-tissue vascular anomalies part 2: vascular malformations. Pediatr Radiol 47(9):1196–1208. 10.1007/s00247-017-3906-x 28779187 10.1007/s00247-017-3906-x

[83] Higgins LJ, Koshy J, Mitchell SE et al (2014) Time-resolved contrast-enhanced MRA (TWIST) with gadofosveset trisodium in the classification of soft-tissue vascular anomalies in the head and neck in children following updated 2014 ISSVA classification: first report on systematic evaluation of MRI and TWIST in a cohort of 47 children. Clin Radiol 71(1):32–39. 10.1016/j.crad.2015.09.006 10.1016/j.crad.2015.09.006 26474946

[84] Acord M, Srinivasan A (2021) Management of venous malformations. Semin Interv Radiol 38(2):215–225. 10.1055/s-0041-1729743 10.1055/s-0041-1729743 PMC8175106 34108809

[85] Cao J, Liu J, Zhang X, Wang Z (2023) A systematic review and network meta-analysis of the effectiveness of sclerotherapy for venous malformation. J Vasc Surg Venous Lymphat Disord 11(1):210-218.e3. 10.1016/j.jvsv.2022.08.004 36179784 10.1016/j.jvsv.2022.08.004

[86] Yamaki T, Nozaki M, Sakurai H, Takeuchi M, Soejima K, Kono T (2008) Prospective randomized efficacy of ultrasound-guided foam sclerotherapy compared with ultrasound-guided liquid sclerotherapy in the treatment of symptomatic venous malformations. J Vasc Surg 47(3):578–584. 10.1016/j.jvs.2007.11.026 18295109 10.1016/j.jvs.2007.11.026

[87] Rodriguez Colon R, Cripps C, Blei F, Sharma S (2023) Surgical treatment of vascular anomalies in the extremities: a single surgeon experience. J Vasc Anom 4(4):e072. 10.1097/JOVA.0000000000000072

[88] Gasparella P, Flucher C, Beqo BP et al (2023) Outcome after surgical treatment of venous malformations of the hand in childhood. J Vasc Surg Venous Lymphat Disord 11(4):793–800. 10.1016/j.jvsv.2023.02.004 36906103 10.1016/j.jvsv.2023.02.004 PMC12433768

[89] Adams DM, Trenor CC, Hammill AM et al (2016) Efficacy and safety of sirolimus in the treatment of complicated vascular anomalies. Pediatrics 137(2):e20153257. 10.1542/peds.2015-3257 26783326 10.1542/peds.2015-3257 PMC4732362

[90] Sarbassov DD, Ali SM, Sengupta S et al (2006) Prolonged rapamycin treatment inhibits mTORC2 assembly and Akt/PKB. Mol Cell 22(2):159–168. 10.1016/j.molcel.2006.03.029 16603397 10.1016/j.molcel.2006.03.029

[91] Nätynki M, Kangas J, Miinalainen I et al (2015) Common and specific effects of TIE2 mutations causing venous malformations. Hum Mol Genet 24(22):6374. 10.1093/HMG/DDV349 26319232 10.1093/hmg/ddv349 PMC4614705

[92] Boscolo E, Limaye N, Huang L et al (2015) Rapamycin improves TIE2-mutated venous malformation in murine model and human subjects. J Clin Invest 125(9):3491. 10.1172/JCI76004 26258417 10.1172/JCI76004 PMC4588237

[93] Si Y, Huang J, Li X et al (2020) AKT/FOXO1 axis links cross-talking of endothelial cell and pericyte in TIE2-mutated venous malformations. Cell Commun Signal 18(1):139. 10.1186/s12964-020-00606-w 32867785 10.1186/s12964-020-00606-w PMC7457504

[94] Cai Y, Schrenk S, Goines J, Davis GE, Boscolo E (2019) Constitutive active mutant TIE2 induces enlarged vascular lumen formation with loss of apico-basal polarity and pericyte recruitment. Sci Rep 9(1):12352. 10.1038/s41598-019-48854-2 31451744 10.1038/s41598-019-48854-2 PMC6710257

[95] Li X, Cai Y, Goines J et al (2019) Ponatinib combined with rapamycin causes regression of murine venous malformation. Arterioscler Thromb Vasc Biol 39(3):496. 10.1161/ATVBAHA.118.312315 30626204 10.1161/ATVBAHA.118.312315 PMC6392210

[96] Hammer J, Seront E, Duez S et al (2018) Sirolimus is efficacious in treatment for extensive and/or complex slow-flow vascular malformations: a monocentric prospective phase II study. Orphanet J Rare Dis 13(1):191. 10.1186/s13023-018-0934-z 30373605 10.1186/s13023-018-0934-z PMC6206885

[97] Maruani A, Tavernier E, Boccara O et al (2021) Sirolimus (rapamycin) for slow-flow malformations in children: the observational-phase randomized clinical PERFORMUS trial. JAMA Dermatol 157(11):1289–1298. 10.1001/jamadermatol.2021.3459 34524406 10.1001/jamadermatol.2021.3459 PMC8444064

[98] Wang G, Lu W, Zhu Y, Wang C, Yang X (2025) Effectiveness and safety of sirolimus in the treatment of venous malformations: a meta-analysis of prospective studies. J Vasc Surg Venous Lymphat Disord 13(6):102284. 10.1016/j.jvsv.2025.102284 40619100 10.1016/j.jvsv.2025.102284 PMC12340385

[99] André F, Ciruelos E, Rubovszky G et al (2019) Alpelisib for PIK3CA-mutated, hormone receptor-positive advanced breast cancer. N Engl J Med 380(20):1929–1940. 10.1056/NEJMoa1813904 31091374 10.1056/NEJMoa1813904

[100] Sterba M, Pokorna P, Faberova R et al (2023) Targeted treatment of severe vascular malformations harboring PIK3CA and TEK mutations with alpelisib is highly effective with limited toxicity. Sci Rep 13(1):10499. 10.1038/s41598-023-37468-4 37380669 10.1038/s41598-023-37468-4 PMC10307862

[101] Remy A, Tran TH, Dubois J et al (2022) Repurposing alpelisib, an anti-cancer drug, for the treatment of severe TIE2-mutated venous malformations: preliminary pharmacokinetics and pharmacodynamic data. Pediatr Blood Cancer 69(10):e29897. 10.1002/pbc.29897 35876545 10.1002/pbc.29897

[102] Medina-Leyte DJ, Domínguez-Pérez M, Mercado I, Villarreal-Molina MT, Jacobo-Albavera L (2020) Use of human umbilical vein endothelial cells (HUVEC) as a model to study cardiovascular disease: a review. Appl Sci 10(3):3. 10.3390/app10030938

[103] Aw WY, Cho C, Wang H et al (2023) Microphysiological model of PIK3CA-driven vascular malformations reveals a role of dysregulated Rac1 and mTORC1/2 in lesion formation. Sci Adv 9(7):eade8939. 10.1126/sciadv.ade8939 36791204 10.1126/sciadv.ade8939 PMC9931220

[104] Lazovic B, Nguyen HT, Ansarizadeh M et al (2024) Human iPSC and CRISPR targeted gene knock-in strategy for studying the somatic TIE2L914F mutation in endothelial cells. Angiogenesis 27(3):523–542. 10.1007/s10456-024-09925-9 38771392 10.1007/s10456-024-09925-9 PMC11303492

[105] Abrahimi P, Chang WG, Kluger MS et al (2015) Efficient gene disruption in cultured primary human endothelial cells by CRISPR/Cas9. Circ Res 117(2):121–128. 10.1161/CIRCRESAHA.117.306290 25940550 10.1161/CIRCRESAHA.117.306290 PMC4490936

[106] Shi Y, Inoue H, Wu JC, Yamanaka S (2017) Induced pluripotent stem cell technology: a decade of progress. Nat Rev Drug Discov 16(2):115–130. 10.1038/nrd.2016.245 27980341 10.1038/nrd.2016.245 PMC6416143

[107] Patsch C, Challet-Meylan L, Thoma EC et al (2015) Generation of vascular endothelial and smooth muscle cells from human pluripotent stem cells. Nat Cell Biol 17(8):994–1003. 10.1038/ncb3205 26214132 10.1038/ncb3205 PMC4566857

[108] Wang K, Lin RZ, Hong X et al (2020) Robust differentiation of human pluripotent stem cells into endothelial cells via temporal modulation of ETV2 with modified mRNA. Sci Adv 6(30):eaba7606. 10.1126/sciadv.aba7606 32832668 10.1126/sciadv.aba7606 PMC7439318

[109] Luo AC, Wang J, Wang K et al (2024) A streamlined method to generate endothelial cells from human pluripotent stem cells via transient doxycycline-inducible ETV2 activation. Angiogenesis 27(4):779–795. 10.1007/s10456-024-09937-5 38969874 10.1007/s10456-024-09937-5 PMC11577265

[110] Schrenk S, Goines J, Boscolo E (2020) A patient-derived xenograft model for venous malformation. J Vis Exp JoVE. 10.3791/61501 32597867 10.3791/61501 PMC7457366

[111] Du Z, Ma HL, Zhang ZY, Zheng JW, Wang YA (2018) Transgenic expression of a venous malformation related mutation, TIE2-R849W, significantly induces multiple malformations of zebrafish. Int J Med Sci 15(4):385–394. 10.7150/ijms.23054 29511374 10.7150/ijms.23054 PMC5835709

[112] Bell LM, Holm A, Matysiak U et al (2022) Functional assessment of two variants of unknown significance in TEK by endothelium-specific expression in zebrafish embryos. Hum Mol Genet 31(1):10–17. 10.1093/hmg/ddab196 10.1093/hmg/ddab196 34254124

[113] Brunsdon H, Wang N, Raredon MSB, Madsen RR, Semple RK, Patton EE (2026) PIK3CA-related overgrowth spectrum (PROS) zebrafish models reveal pan-lineage developmental dysregulation. eLife. 10.7554/eLife.110896.1

[114] di Blasio L, Puliafito A, Gagliardi PA et al (2018) PI3K/mTOR inhibition promotes the regression of experimental vascular malformations driven by PIK3CA-activating mutations. Cell Death Dis 9(2):45. 10.1038/s41419-017-0064-x 29352118 10.1038/s41419-017-0064-x PMC5833448

[115] Kobialka P, Sabata H, Vilalta O et al (2022) The onset of PI3K-related vascular malformations occurs during angiogenesis and is prevented by the AKT inhibitor miransertib. EMBO Mol Med 14(7):e15619. 10.15252/emmm.202115619 35695059 10.15252/emmm.202115619 PMC9260211

[116] Petkova M, Kraft M, Stritt S et al (2023) Immune-interacting lymphatic endothelial subtype at capillary terminals drives lymphatic malformation. J Exp Med 220(4):e20220741. 10.1084/jem.20220741 36688917 10.1084/jem.20220741 PMC9884640

[117] Kraft M, Schoofs H, Petkova M et al (2025) Angiopoietin–TIE2 feedforward circuit promotes PIK3CA-driven venous malformations. Nat Cardiovasc Res 4(7):801–820. 10.1038/s44161-025-00655-9 40410415 10.1038/s44161-025-00655-9 PMC12259471

[118] Bischoff LJ, Schrenk S, Soroko K et al (2025) Expression of mutant TIE2 p.L914F during mouse development causes embryonic lethality and defects in vascular remodeling. Dev Dyn. 10.1002/dvdy.70087 41074681 10.1002/dvdy.70087 PMC12853423

[119] Bischoff LJ, Sherpa C, Schrenk S, Boscolo E (2026) Constitutive, mosaic expression of TIE2 p.L914F during mouse development causes venous malformation. FASEB J 40(9):e71862. 10.1096/fj.202600444R 42059767 10.1096/fj.202600444R PMC13159068

[120] Schnürch H, Risau W (1993) Expression of tie-2, a member of a novel family of receptor tyrosine kinases, in the endothelial cell lineage. Development 119(3):957–968. 10.1242/dev.119.3.957 8187650 10.1242/dev.119.3.957

[121] Kisanuki YY, Hammer RE, Miyazaki J, Williams SC, Richardson JA, Yanagisawa M (2001) Tie2-Cre transgenic mice: a new model for endothelial cell-lineage analysis in vivo. Dev Biol 230(2):230–242. 10.1006/dbio.2000.0106 11161575 10.1006/dbio.2000.0106

[122] Puri MC, Bernstein A (2003) Requirement for the TIE family of receptor tyrosine kinases in adult but not fetal hematopoiesis. Proc Natl Acad Sci U S A 100(22):12753–12758. 10.1073/pnas.2133552100 14530387 10.1073/pnas.2133552100 PMC240690

[123] Tang Y, Harrington A, Yang X, Friesel RE, Liaw L (2010) The contribution of the Tie2+ lineage to primitive and definitive hematopoietic cells. Genes N Y N 2000 48(9):563–567. 10.1002/dvg.20654 10.1002/dvg.20654 PMC2944906 20645309

[124] Arai F, Hirao A, Ohmura M et al (2004) Tie2/angiopoietin-1 signaling regulates hematopoietic stem cell quiescence in the bone marrow niche. Cell 118(2):149–161. 10.1016/j.cell.2004.07.004 15260986 10.1016/j.cell.2004.07.004

[125] Venneri MA, De Palma M, Ponzoni M et al (2007) Identification of proangiogenic TIE2-expressing monocytes (TEMs) in human peripheral blood and cancer. Blood 109(12):5276–5285. 10.1182/blood-2006-10-053504 17327411 10.1182/blood-2006-10-053504

[126] Barton WA, Tzvetkova-Robev D, Miranda EP et al (2006) Crystal structures of the Tie2 receptor ectodomain and the angiopoietin-2–Tie2 complex. Nat Struct Mol Biol 13(6):524–532. 10.1038/nsmb1101 16732286 10.1038/nsmb1101

[127] Fiedler U, Krissl T, Koidl S et al (2003) Angiopoietin-1 and angiopoietin-2 share the same binding domains in the Tie-2 receptor involving the first Ig-like loop and the epidermal growth factor-like repeats *. J Biol Chem 278(3):1721–1727. 10.1074/jbc.M208550200 12427764 10.1074/jbc.M208550200

[128] Jo G, Bae J, Hong HJ et al (2021) Structural insights into the clustering and activation of Tie2 receptor mediated by Tie2 agonistic antibody. Nat Commun 12(1):6287. 10.1038/s41467-021-26620-1 34725372 10.1038/s41467-021-26620-1 PMC8560823

[129] Shewchuk LM, Hassell AM, Ellis B et al (2000) Structure of the Tie2 RTK Domain: Self-Inhibition by the Nucleotide Binding Loop, Activation Loop, and C-Terminal Tail. Structure 8(11):1105–1113. 10.1016/S0969-2126(00)00516-5 11080633 10.1016/s0969-2126(00)00516-5

[130] Yancopoulos GD, Davis S, Gale NW, Rudge JS, Wiegand SJ, Holash J (2000) Vascular-specific growth factors and blood vessel formation. Nature 407(6801):242–248. 10.1038/35025215 11001067 10.1038/35025215

[131] Yuan HT, Khankin EV, Karumanchi SA, Parikh SM (2009) Angiopoietin 2 is a partial agonist/antagonist of Tie2 signaling in the endothelium. Mol Cell Biol 29(8):2011–2022. 10.1128/MCB.01472-08 19223473 10.1128/MCB.01472-08 PMC2663314

[132] Davis S, Papadopoulos N, Aldrich TH et al (2003) Angiopoietins have distinct modular domains essential for receptor binding, dimerization and superclustering. Nat Struct Biol 10(1):38–44. 10.1038/nsb880 12469114 10.1038/nsb880

[133] Cho CH, Kammerer RA, Lee HJ et al (2004) COMP-Ang1: a designed angiopoietin-1 variant with nonleaky angiogenic activity. Proc Natl Acad Sci U S A 101(15):5547–5552. 10.1073/pnas.0307574101 15060279 10.1073/pnas.0307574101 PMC397420

[134] Yu X, Seegar TCM, Dalton AC et al (2013) Structural basis for angiopoietin-1–mediated signaling initiation. Proc Natl Acad Sci U S A 110(18):7205–7210. 10.1073/pnas.1216890110 23592718 10.1073/pnas.1216890110 PMC3645508

[135] Seegar TCM, Eller B, Tzvetkova-Robev D et al (2010) Tie1-Tie2 interactions mediate functional differences between angiopoietin ligands. Mol Cell 37(5):643–655. 10.1016/j.molcel.2010.02.007 20227369 10.1016/j.molcel.2010.02.007 PMC2841065

[136] Teichert-Kuliszewska K, Maisonpierre PC, Jones N et al (2001) Biological action of angiopoietin-2 in a fibrin matrix model of angiogenesis is associated with activation of Tie2. Cardiovasc Res 49(3):659–670. 10.1016/s0008-6363(00)00231-5 11166279 10.1016/s0008-6363(00)00231-5

[137] Davis S, Aldrich TH, Jones PF et al (1996) Isolation of angiopoietin-1, a ligand for the TIE2 receptor, by secretion-trap expression cloning. Cell 87(7):1161–1169. 10.1016/S0092-8674(00)81812-7 8980223 10.1016/s0092-8674(00)81812-7

[138] Valenzuela DM, Griffiths JA, Rojas J et al (1999) Angiopoietins 3 and 4: diverging gene counterparts in mice and humans. Proc Natl Acad Sci U S A 96(5):1904–1909. 10.1073/pnas.96.5.1904 10051567 10.1073/pnas.96.5.1904 PMC26709

[139] Kontos CD, Cha EH, York JD, Peters KG (2002) The endothelial receptor tyrosine kinase Tie1 activates phosphatidylinositol 3-kinase and Akt to inhibit apoptosis. Mol Cell Biol 22(6):1704–1713. 10.1128/MCB.22.6.1704-1713.2002 11865050 10.1128/MCB.22.6.1704-1713.2002 PMC135589

[140] Yuan HT, Venkatesha S, Chan B et al (2007) Activation of the orphan endothelial receptor Tie1 modifies Tie2-mediated intracellular signaling and cell survival. FASEB J 21(12):3171–3183. 10.1096/fj.07-8487com 17504972 10.1096/fj.07-8487com

[141] Korhonen EA, Lampinen A, Giri H et al (2016) Tie1 controls angiopoietin function in vascular remodeling and inflammation. J Clin Invest 126(9):3495–3510. 10.1172/JCI84923 27548530 10.1172/JCI84923 PMC5004934

[142] Savant S, La Porta S, Budnik A et al (2015) The orphan receptor Tie1 controls angiogenesis and vascular remodeling by differentially regulating Tie2 in tip and stalk cells. Cell Rep 12(11):1761–1773. 10.1016/j.celrep.2015.08.024 26344773 10.1016/j.celrep.2015.08.024 PMC6309948

[143] Zhang X, Ishibashi M, Kitatani K et al (2020) Potential of Tyrosine Kinase Receptor TIE-1 as novel therapeutic target in high-PI3K-expressing ovarian cancer. Cancers 12(6):1705. 10.3390/cancers12061705 32604863 10.3390/cancers12061705 PMC7352248

[144] Baldwin HS, Davis MJ, Harmelink C, Qu X (2025) TIE1-dependent lymphatic vascular remodeling is mediated by its second tyrosine kinase domain. Dev Camb Engl 152(13):dev204469. 10.1242/dev.204469 10.1242/dev.204469 PMC12268172 40539480

[145] Brouillard P, Murtomäki A, Leppänen VM et al (2024) Loss-of-function mutations of the TIE1 receptor tyrosine kinase cause late-onset primary lymphedema. J Clin Invest. 134(14):e173586. 10.1172/JCI173586 38820174 10.1172/JCI173586 PMC11245153

[146] Anisimov A, Lackman MH, Augustin HG, Mervaala E, Alitalo K, Karaman S (2026) Deletion of the angiopoietin receptor Tie2 enhances proliferation and sprouting of cardiac endothelial cells. Angiogenesis 29(2):13. 10.1007/s10456-025-10028-2 41563573 10.1007/s10456-025-10028-2 PMC12823625

[147] Fachinger G, Deutsch U, Risau W (1999) Functional interaction of vascular endothelial-protein-tyrosine phosphatase with the Angiopoietin receptor Tie-2. Oncogene 18(43):5948–5953. 10.1038/sj.onc.1202992 10557082 10.1038/sj.onc.1202992

[148] Baluk P, Shirakura K, Vestweber D, McDonald DM (2024) Heterogeneity of endothelial VE-PTP downstream polarization, Tie2 activation, junctional claudin-5, and permeability in the aorta and vena cava. Cell Tissue Res 395(1):81–103. 10.1007/s00441-023-03844-9 38032480 10.1007/s00441-023-03844-9 PMC10774230

[149] Shen J, Frye M, Lee BL et al (2014) Targeting VE-PTP activates TIE2 and stabilizes the ocular vasculature. J Clin Invest 124(10):4564–4576. 10.1172/JCI74527 25180601 10.1172/JCI74527 PMC4191011

[150] Souma T, Thomson BR, Heinen S et al (2018) Context-dependent functions of angiopoietin 2 are determined by the endothelial phosphatase VEPTP. Proc Natl Acad Sci U S A 115(6):1298–1303. 10.1073/pnas.1714446115 29358379 10.1073/pnas.1714446115 PMC5819405

[151] Zheng YY, Hua C, Lin XX (2025) PI3K/AKT/mTOR axis in vascular malformations: from molecular insights to targeted clinical trials. Orphanet J Rare Dis 20:624. 10.1186/s13023-025-04115-2 41430313 10.1186/s13023-025-04115-2 PMC12723890

[152] Katso R, Okkenhaug K, Ahmadi K, White S, Timms J, Waterfield MD (2001) Cellular function of phosphoinositide 3-kinases: implications for development, homeostasis, and cancer. Annu Rev Cell Dev Biol 17:615–675. 10.1146/annurev.cellbio.17.1.615 11687500 10.1146/annurev.cellbio.17.1.615

[153] Chu N, Salguero AL, Liu AZ et al (2018) Akt kinase activation mechanisms revealed using protein semisynthesis. Cell 174(4):897-907.e14. 10.1016/j.cell.2018.07.003 30078705 10.1016/j.cell.2018.07.003 PMC6139374

[154] Manning BD, Cantley LC (2007) AKT/PKB signaling: navigating downstream. Cell 129(7):1261–1274. 10.1016/j.cell.2007.06.009 17604717 10.1016/j.cell.2007.06.009 PMC2756685

[155] Jones N, Master Z, Jones J et al (1999) Identification of Tek/Tie2 binding partners. Binding to a multifunctional docking site mediates cell survival and migration. J Biol Chem 274(43):30896–30905 10521483 10.1074/jbc.274.43.30896

[156] Daly C, Wong V, Burova E et al (2004) Angiopoietin-1 modulates endothelial cell function and gene expression via the transcription factor FKHR (FOXO1). Genes Dev 18(9):1060–1071. 10.1101/gad.1189704 15132996 10.1101/gad.1189704 PMC406295

[157] DeBusk LM, Hallahan DE, Lin PC (2004) Akt is a major angiogenic mediator downstream of the Ang1/Tie2 signaling pathway. Exp Cell Res 298(1):167–177. 10.1016/j.yexcr.2004.04.013 15242771 10.1016/j.yexcr.2004.04.013

[158] Kim I, Kim HG, So JN, Kim JH, Kwak HJ, Koh GY (2000) Angiopoietin-1 regulates endothelial cell survival through the phosphatidylinositol 3′-kinase/Akt signal transduction pathway. Circ Res 86(1):24–29. 10.1161/01.RES.86.1.24 10625301 10.1161/01.res.86.1.24

[159] Kanda S, Miyata Y, Mochizuki Y, Matsuyama T, Kanetake H (2005) Angiopoietin 1 is mitogenic for cultured endothelial cells. Cancer Res 65(15):6820–6827. 10.1158/0008-5472.CAN-05-0522 16061664 10.1158/0008-5472.CAN-05-0522

[160] Guo YJ, Pan WW, Liu SB, Shen ZF, Xu Y, Hu LL (2020) ERK/MAPK signalling pathway and tumorigenesis. Exp Ther Med 19(3):1997–2007. 10.3892/etm.2020.8454 32104259 10.3892/etm.2020.8454 PMC7027163

[161] Fukuhara S, Sako K, Minami T et al (2008) Differential function of Tie2 at cell–cell contacts and cell–substratum contacts regulated by angiopoietin-1. Nat Cell Biol 10(5):513–526. 10.1038/ncb1714 18425120 10.1038/ncb1714

[162] Echavarria R, Hussain SNA (2013) Regulation of angiopoietin-1/Tie-2 receptor signaling in endothelial cells by dual-specificity phosphatases 1, 4, and 5. J Am Heart Assoc Cardiovasc Cerebrovasc Dis 2(6):e000571. 10.1161/JAHA.113.000571 10.1161/JAHA.113.000571 PMC3886752 24308939

[163] Kim I, Ryu YS, Kwak HJ et al (2002) EphB ligand, ephrinB2, suppresses the VEGF- and angiopoietin 1-induced Ras/mitogen-activated protein kinase pathway in venous endothelial cells. FASEB J 16(9):1126–1128. 10.1096/FJ.01-0805FJE 12039842 10.1096/fj.01-0805fje

[164] Dori Y, Smith C, Pinto E et al (2020) Severe lymphatic disorder resolved with MEK inhibition in a patient with Noonan syndrome and SOS1 mutation. Pediatrics 146(6):e20200167. 10.1542/peds.2020-0167 33219052 10.1542/peds.2020-0167

[165] Sun Y, Gu H, Zhang B et al (2025) Trametinib treatment for early-stage extracranial arteriovenous malformations: a multicenter, prospective, single-arm pilot study. J Am Acad Dermatol 93(5):1261–1267. 10.1016/j.jaad.2025.07.033 40683355 10.1016/j.jaad.2025.07.033

[166] Chislock EM, Ring C, Pendergast AM (2013) Abl kinases are required for vascular function, Tie2 expression, and angiopoietin-1-mediated survival. Proc Natl Acad Sci U S A 110(30):12432–12437. 10.1073/PNAS.1304188110/-/DCSUPPLEMENTAL 23840065 10.1073/pnas.1304188110 PMC3725093

[167] Raimondi C, Fantin A, Lampropoulou A, Denti L, Chikh A, Ruhrberg C (2014) Imatinib inhibits VEGF-independent angiogenesis by targeting neuropilin 1–dependent ABL1 activation in endothelial cells. J Exp Med 211(6):1167–1183. 10.1084/jem.20132330 24863063 10.1084/jem.20132330 PMC4042645

[168] Schumacher JA, Wright ZA, Rufin Florat D et al (2024) SH2 domain protein E and ABL signaling regulate blood vessel size. PLoS Genet 20(1):e1010851. 10.1371/journal.pgen.1010851 38190417 10.1371/journal.pgen.1010851 PMC10798624

[169] Tang W, Li Y, Boscolo E, Kohane DS, Cullion K (2026) Polymeric rapamycin nanoparticles encapsulating ponatinib cause regression of venous malformations in mice. Sci Transl Med 18(848):eaeb7597. 10.1126/scitranslmed.aeb7597 42090479 10.1126/scitranslmed.aeb7597 PMC13383109

[170] Dumont DJ, Gradwohl G, Fong GH et al (1994) Dominant-negative and targeted null mutations in the endothelial receptor tyrosine kinase, tek, reveal a critical role in vasculogenesis of the embryo. Genes Dev 8(16):1897–1909. 10.1101/gad.8.16.1897 7958865 10.1101/gad.8.16.1897

[171] Sato TN, Tozawa Y, Deutsch U et al (1995) Distinct roles of the receptor tyrosine kinases Tie-1 and Tie-2 in blood vessel formation. Nature 376(6535):70–74. 10.1038/376070a0 7596437 10.1038/376070a0

[172] Qu X, Tompkins K, Batts LE, Puri M, Baldwin HS (2010) Abnormal embryonic lymphatic vessel development in Tie1 hypomorphic mice. Dev Camb Engl 137(8):1285–1295. 10.1242/dev.043380 10.1242/dev.043380 PMC2847464 20223757

[173] Chu M, Li T, Shen B et al (2016) Angiopoietin receptor Tie2 is required for vein specification and maintenance via regulating COUP-TFII. eLife 5:e21032. 10.7554/eLife.21032 28005008 10.7554/eLife.21032 PMC5218530

[174] Li-Villarreal N, Wong RLY, Garcia MD et al (2022) FOXO1 represses sprouty 2 and sprouty 4 expression to promote arterial specification and vascular remodeling in the mouse yolk sac. Development 149(7):dev200131. 10.1242/dev.200131 35297995 10.1242/dev.200131 PMC8995087

[175] Cao X, Li T, Xu B et al (2023) Endothelial TIE1 restricts angiogenic sprouting to coordinate vein assembly in synergy with its homologue TIE2. Arterioscler Thromb Vasc Biol 43(8):e323–e338. 10.1161/ATVBAHA.122.318860 37317851 10.1161/ATVBAHA.122.318860

[176] Suri C, Jones PF, Patan S et al (1996) Requisite role of angiopoietin-1, a ligand for the TIE2 receptor, during embryonic angiogenesis. Cell 87(7):1171–1180. 10.1016/S0092-8674(00)81813-9 8980224 10.1016/s0092-8674(00)81813-9

[177] Jeansson M, Gawlik A, Anderson G et al (2011) Angiopoietin-1 is essential in mouse vasculature during development and in response to injury. J Clin Invest 121(6):2278–2289. 10.1172/JCI46322 21606590 10.1172/JCI46322 PMC3104773

[178] Gale NW, Thurston G, Hackett SF et al (2002) Angiopoietin-2 is required for postnatal angiogenesis and lymphatic patterning, and only the latter role is rescued by Angiopoietin-1. Dev Cell 3(3):411–423. 10.1016/S1534-5807(02)00217-4 12361603 10.1016/s1534-5807(02)00217-4

[179] Maisonpierre PC, Suri C, Jones PF et al (1997) Angiopoietin-2, a natural antagonist for Tie2 that disrupts in vivo angiogenesis. Science 277(5322):55–60. 10.1126/science.277.5322.55 9204896 10.1126/science.277.5322.55

[180] Wong AL, Haroon ZA, Werner S, Dewhirst MW, Greenberg CS, Peters KG (1997) Tie2 expression and phosphorylation in angiogenic and quiescent adult tissues. Circ Res 81(4):567–574. 10.1161/01.RES.81.4.567 9314838 10.1161/01.res.81.4.567

[181] Papapetropoulos A, Fulton D, Mahboubi K et al (2000) Angiopoietin-1 inhibits endothelial cell apoptosis via the Akt/survivin pathway. J Biol Chem 275(13):9102–9105. 10.1074/jbc.275.13.9102 10734041 10.1074/jbc.275.13.9102

[182] Tsigkos S, Zhou Z, Kotanidou A et al (2006) Regulation of Ang2 release by PTEN/PI3-kinase/Akt in lung microvascular endothelial cells. J Cell Physiol 207(2):506–511. 10.1002/jcp.20592 16447257 10.1002/jcp.20592

[183] Gamble JR, Drew J, Trezise L et al (2000) Angiopoietin-1 is an antipermeability and anti-inflammatory agent in vitro and targets cell junctions. Circ Res 87(7):603–607. 10.1161/01.RES.87.7.603/FORMAT/EPUB 11009566 10.1161/01.res.87.7.603

[184] Winkler F, Kozin SV, Tong RT et al (2004) Kinetics of vascular normalization by VEGFR2 blockade governs brain tumor response to radiation: role of oxygenation, angiopoietin-1, and matrix metalloproteinases. Cancer Cell 6(6):553–563. 10.1016/j.ccr.2004.10.011 15607960 10.1016/j.ccr.2004.10.011

[185] Felcht M, Luck R, Schering A et al (2012) Angiopoietin-2 differentially regulates angiogenesis through TIE2 and integrin signaling. J Clin Invest 122(6):1991–2005. 10.1172/JCI58832 22585576 10.1172/JCI58832 PMC3366398

[186] Schonning MJ, Koh S, Sun RW et al (2021) Venous malformation vessels are improperly specified and hyperproliferative. PLoS ONE. 10.1371/journal.pone.0252342 34043714 10.1371/journal.pone.0252342 PMC8158993

[187] Lee HW, Shin JH, Simons M (2022) Flow goes forward and cells step backward: endothelial migration. Exp Mol Med 54(6):711–719. 10.1038/s12276-022-00785-1 35701563 10.1038/s12276-022-00785-1 PMC9256678

[188] Lee HW, Xu Y, He L et al (2021) The role of venous endothelial cells in developmental and pathologic angiogenesis. Circulation 144(16):1308. 10.1161/CIRCULATIONAHA.121.054071 34474596 10.1161/CIRCULATIONAHA.121.054071 PMC9153651

[189] Ansarizadeh M, Lazovic B, Sarmadian Z, Singh A, Hicks R, Eklund L (2026) A human endothelial and adipose stem cell-based co-culture model for venous malformations. Angiogenesis 29(3):30. 10.1007/s10456-026-10045-9 42070175 10.1007/s10456-026-10045-9 PMC13136223

[190] Koh W, Mahan RD, Davis GE (2008) Cdc42- and Rac1-mediated endothelial lumen formation requires Pak2, Pak4 and Par3, and PKC-dependent signaling. J Cell Sci 121(7):989–1001. 10.1242/jcs.020693 18319301 10.1242/jcs.020693

[191] Barry DM, Koo Y, Norden PR et al (2016) Rasip1-mediated Rho GTPase signaling regulates blood vessel tubulogenesis via nonmuscle myosin II. Circ Res 119(7):810–826. 10.1161/CIRCRESAHA.116.309094 27486147 10.1161/CIRCRESAHA.116.309094 PMC5026621

[192] Laviña B, Castro M, Niaudet C et al (2018) Defective endothelial cell migration in the absence of Cdc42 leads to capillary-venous malformations. Development 145(13):dev161182. 10.1242/dev.161182 29853619 10.1242/dev.161182

[193] Castro M, Laviña B, Ando K et al (2019) CDC42 deletion elicits cerebral vascular malformations via increased MEKK3-dependent KLF4 expression. Circ Res 124(8):1240–1252. 10.1161/CIRCRESAHA.118.314300 30732528 10.1161/CIRCRESAHA.118.314300

[194] García-Cardeña G, Comander J, Anderson KR, Blackman BR, Gimbrone MA (2001) Biomechanical activation of vascular endothelium as a determinant of its functional phenotype. Proc Natl Acad Sci U S A 98(8):4478–4485. 10.1073/pnas.071052598 11296290 10.1073/pnas.071052598 PMC31860

[195] Baeyens N, Nicoli S, Coon BG et al (2015) Vascular remodeling is governed by a VEGFR3-dependent fluid shear stress set point. eLife 4:e04645. 10.7554/eLife.04645 25643397 10.7554/eLife.04645 PMC4337723

[196] Baeyens N, Bandyopadhyay C, Coon BG, Yun S, Schwartz MA (2016) Endothelial fluid shear stress sensing in vascular health and disease. J Clin Invest 126(3):821–828. 10.1172/JCI83083 26928035 10.1172/JCI83083 PMC4767335

[197] Aw WY, Sawhney A, Rathod M et al (2025) Dysfunctional mechanotransduction regulates the progression of PIK3CA-driven vascular malformations. APL Bioeng 9(1):016106. 10.1063/5.0234507 39935869 10.1063/5.0234507 PMC11811908

[198] Lee HJ, Koh GY (2003) Shear stress activates Tie2 receptor tyrosine kinase in human endothelial cells. Biochem Biophys Res Commun 304(2):399–404. 10.1016/s0006-291x(03)00592-8 12711329 10.1016/s0006-291x(03)00592-8

[199] Shirakura K, Baluk P, Nottebaum AF et al (2023) Shear stress control of vascular leaks and atheromas through Tie2 activation by VE-PTP sequestration. EMBO Mol Med 15(4):e16128. 10.15252/emmm.202216128 36740996 10.15252/emmm.202216128 PMC10086590

[200] Chen G, Ren JG, Zhang W et al (2014) Disorganized vascular structures in sporadic venous malformations: a possible correlation with balancing effect between Tie2 and TGF-β. Sci Rep 4:5457. 10.1038/srep05457 24966004 10.1038/srep05457 PMC4071312

[201] Zhu J, Tang Z, Ren J et al (2022) Downregulation of microRNA-21 contributes to decreased collagen expression in venous malformations via transforming growth factor-β/Smad3/microRNA-21 signaling feedback loop. J Vasc Surg Venous Lymphat Disord 10(2):469-481.e2. 10.1016/j.jvsv.2021.08.020 34506963 10.1016/j.jvsv.2021.08.020

[202] Chen GH, Yang JG, Xia HF et al (2022) Endothelial cells induce degradation of ECM through enhanced secretion of MMP14 carried on extracellular vesicles in venous malformation. Cell Tissue Res 389(3):517–530. 10.1007/S00441-022-03657-2/FIGURES/6 35786766 10.1007/s00441-022-03657-2

[203] Zhang R, Han Z, Degos V et al (2016) Persistent infiltration and pro-inflammatory differentiation of monocytes cause unresolved inflammation in brain arteriovenous malformation. Angiogenesis 19(4):451–461. 10.1007/s10456-016-9519-4 27325285 10.1007/s10456-016-9519-4 PMC5029790

[204] Tang AT, Choi JP, Kotzin JJ et al (2017) Endothelial TLR4 and the microbiome drive cerebral cavernous malformations. Nature 545(7654):305–310. 10.1038/nature22075 28489816 10.1038/nature22075 PMC5757866

[205] Lai CC, Nelsen B, Frias-Anaya E et al (2022) Neuroinflammation plays a critical role in cerebral cavernous malformation disease. Circ Res 131(11):909–925. 10.1161/CIRCRESAHA.122.321129 36285625 10.1161/CIRCRESAHA.122.321129 PMC9669201

[206] Liu L, Wang L, Luo J et al (2025) Endothelial GNAQ p.R183Q mutation confers hemoporfin-mediated photodynamic therapy resistance and drives pathological angiogenesis via the angiopoietin-2/TIE2/PI3K/AKT pathway. Front Cell Dev Biol 13:1622961. 10.3389/fcell.2025.1622961 40917747 10.3389/fcell.2025.1622961 PMC12411732

[207] Li L, Castro M, Hongo H et al (2026) TIE2 links MEKK3–KLF2/4 and PI3K signaling in cerebral cavernous malformation. J Exp Med 223(5):e20251374. 10.1084/jem.20251374 41891922 10.1084/jem.20251374 PMC13459978

[208] Banerjee K, Lin Y, Gahn J et al (2023) SMAD4 maintains the fluid shear stress set point to protect against arterial-venous malformations. J Clin Invest 133(18):e168352. 10.1172/JCI168352 37490341 10.1172/JCI168352 PMC10503796

